# The emotional cost of containment: a cross-sectional analysis of treatment effects among informal carers in South Asia during the COVID-19 pandemic

**DOI:** 10.1080/16549716.2025.2504227

**Published:** 2025-06-03

**Authors:** Carol Troy, Anna Tjin, Anna Goodwin, Iracema Leroi, Roger O’Sullivan, Yaohua Chen, on behalf of the CLIC Caregiver Study Group

**Affiliations:** a International Business Administration, International College, Tunghai University, Taichung, Taiwan R.O.C; b Psychological Medicine, Institute of Psychiatry, Psychology & Neuroscience, Kings College London, London, UK; c Global Health Institute, Trinity College Dublin, Dublin, Ireland; d Global Brain Health Institute, Trinity College Dublin, Dublin, Ireland; e Trinity College Institute of Neuroscience, School of Medicine, Trinity College, Dublin, Ireland; f Institute of Public Health, Belfast, Ireland; g The Bamford Centre for Mental Health and Wellbeing, Ulster University, Coleraine, UK; h CHU Lille, Lille Neurosciences & Cognition, UMR-S1172, Degenerative and Vascular Cognitive Disorders, Lille CHU University of Lille, Lille, France

**Keywords:** Jennifer Stewart Williams, Propensity score matching, factor analysis, social isolation, information and communication technology, treatment effects

## Abstract

**Background:**

The COVID-19 pandemic led to government-imposed mobility restrictions, social distancing, and lockdowns, altering the caregiving environment worldwide. In South Asia, it is unknown what aspects of these changes posed significant emotional costs to informal carers, or how such costs can be mitigated in future pandemics.

**Objectives:**

To identify environmental change aspects (dimensions) that posed distinct emotional costs for South Asian carers. To quantify the costs and classify them as persistent, transient, hidden, or insignificant. To propose ways of mitigating carer distress during future pandemics.

**Methods:**

The data came from the Coping with Loneliness, Isolation, and COVID-19 Caregiver survey. Carers (n=454) in Bangladesh (N=123), India (N=116), and Pakistan (N=215) self-reported their experiences before/during the pandemic. The dimensions were extracted from 11 change indicators. A dimension’s emotional costs were its effects on (1) the change in burden frequency relative to pre-pandemic conditions and (2) the during-pandemic burden frequency.

**Results:**

Five factors emerged: social confinement, reduced/missing information on the recipient, loss of connection, restricted visitation rights, and protective clothing. Social confinement (loss of connection) increased both changes in burden frequency and during-pandemic burden frequency, indicating a persistent emotional cost to carers. Restricted visitation rights affected only pandemic burden frequency, indicating a hidden emotional impact.

**Conclusions:**

Social confinement (loss of connection, restricted visitation rights) was emotionally costly because it forced an increase (decrease) in care intensity relative to pre-pandemic levels. Through enhanced counseling and emotional support services, South Asian public health systems can alleviate carers’ private suffering during normal times and future crises.

## Paper context

- **Main findings:** During the COVID-19 pandemic, government containment policies changed the caregiving environment, posing emotional costs to South Asian carers.
- **Added knowledge:** The highest emotional costs resulted from undesirable increases and decreases in caregiving intensity relative to the carer’s normal pre-pandemic level.
- **Global health impact for policy and action:** Carer support strategies embedded in local cultural norms, socioeconomic realities, and healthcare systems can enhance the sustainability of informal care in normal times and during crises.

## Background

During the early stages of the COVID-19 pandemic, governments throughout the globe instituted public health containment policies, including mobility restrictions, international travel bans, social distancing, event cancellations, school and work closures, mask mandates, and lockdowns [1–5]. These measures were enforced by institutions and businesses [6,7], particularly at the peak of the pandemic [8,9]. Although the policies achieved some successes in limiting disease transmission [10,11], they changed the caregiving environment in unintended ways. This had consequences for informal carers [12–15], the individuals who deliver unpaid assistance and support to family, friends, and loved ones. The containment measures upended established care routines in numerous households [16,17]. Needed caregiving inputs, such as medical care, daycare services, and informal assistance (e.g. from friends or family) became scarce [8,18], while demanded outputs (e.g. time spent with the recipient) increased [19]. Disruptions to personal lives (e.g. job losses and school closures) spilled over into caregiving, posing additional obstacles, particularly for women [20,21]. Carers adapted to these environmental changes by redoubling their efforts to meet the new demands without compromising quality [22]. For some carers, the adaptations brought psychological impacts beyond the baseline pre-pandemic levels, including more frequent feelings of burden. Thus, the COVID-19 containment strategies adopted by governments posed ‘emotional costs’ for carers. The caregiving literature defines emotional cost as ‘the emotional value a subject pays, in terms of suffering and discomfort, when faced with extreme or traumatic situations’ [23]. Since national health care services throughout the world rely on informal carers [24], recognizing the emotional costs they face is essential to evaluating policies and ensuring the sustainability of informal care systems. This paper will focus on assessing the emotional costs of the COVID-19 pandemic containment measures.

### The pandemic caregiving environment in low- and high-income regions

At the outset of the pandemic, countries worldwide adopted similar containment policies, but implemented them differently, with important consequences for informal carers. In the EU and US, COVID-19 restrictions were shaped by federated governance structures. However, the EU’s response was faster and more cohesive: all 27 members enacted lockdowns by 19 March, compared to just two US states, and more members adopted the strictest lockdowns [25,26]. Mask mandates, mobility restrictions, and internal border controls were implemented more uniformly across the EU [27–29], under the coordination of institutions like the European Commission and the ECDC. In the US, decentralized health infrastructure, lack of national caregiver strategies and support services [30], political polarization [31], and inconsistent public messaging [32] produced a fragmented response. For informal carers, especially women balancing paid work and caregiving, this patchwork response created anxiety, uncertainty, and exhaustion [30,33].

Stress among informal carers in East Asian countries (e.g. China, South Korea, Japan, and Taiwan) was mitigated by the centralized and technologically supported strategies of their governments. Like the EU, these countries promptly imposed mobility restrictions and border controls. By late March, all EU members except Ireland had barred non-essential non-EU travellers [34]; likewise, in late March and early April, China, Japan, and Taiwan implemented near-total bans, and South Korea established 14-day quarantines [35–38]. East Asian countries adopted digital technologies for contact tracing, surveillance, and supply distribution [39]. Moreover, higher levels of government trust − 95.4% in China, 51.3% in South Korea, 43.4% in Japan, and 51.8% in Taiwan, *versus* 30.3% in the EU and 31.0% in the US – boosted compliance and reduced the psychological toll associated with rule ambiguity and public dissent [40]. East Asian carers faced isolation and increased workloads, but often within clearer and more predictable policy environments.

Despite differences in execution, the EU, US, and East Asian strategies all relied on costly interventions, e.g. economic relief to businesses and citizens [41–48] and emergency funding for health systems and infrastructure [31,49–55]. These programs offered some protection for carers in high-income countries. By contrast, low- and middle-income countries (LMICs), following WHO guidance [56] and mimicking the strategies of first-affected countries like China and Italy (e.g. lockdowns and mobility restrictions) [57], lacked the resources to offer equivalent support. Their standardized, top-down containment policies could not accommodate socioeconomic realities such as informal labor markets, overcrowding, and weak health infrastructure [57]. For informal carers in these settings, especially in South Asia, the result was emotional hardship: heightened anxiety, burnout, and a sense of helplessness in the face of mounting demands and limited institutional support.

### The pandemic caregiving environment in South Asia

Three South Asian countries, Bangladesh, India, and Pakistan, exemplified the contradictions of COVID-19 containment in LMICs. Medical resources were relatively scarce: pre-pandemic, South Asia had fewer than half the per capita number of doctors and hospital beds [58] typical in Western countries (e.g. the UK). During the pandemic, healthcare became even less accessible, and by many measures, the quality declined [59]. Fear of contagion often caused patients to avoid hospital visits [2,5]. Some facilities were reserved for COVID-19 patients, further limiting doctor availability [60–62]. Carer access to medical services was therefore severely disrupted.

Lockdowns and closures had detrimental effects on caregiving support services. In a qualitative study of caregiving to persons with dementia, Vaitheswaran et al. [63] found that pre-pandemic, Indian carers had formal and informal support networks, which dwindled during the pandemic. Daycare services were often suspended [64,65], depriving carers of respite and recipients of medical services (e.g. physical rehabilitation). Mobility restrictions also limited assistance from non-cohabiting family members [18], exacerbating the strain on the carer.

During the initial pandemic period, there was a global shortage of Personal Protective Equipment (PPE) [3]. In South Asia, the needs of hospital staff were prioritized. Volunteers and low-paid carers such as community health workers [5,66] struggled to access PPE, often resorting to hand-made substitutes. Inadequate access to PPE heightened stress and fears of COVID-19 exposure.

### The benefits of this study

By examining informal carers in South Asia, this paper will address a research gap, as LMICs like Bangladesh, India, and Pakistan are underrepresented in the literature favoring high-income regions. Furthermore, the study will provide a window on three societies in rapid transition. The caregiving environments in these countries are similar in terms of history, cultural heritage, geography, and socio-economic characteristics [58]. In the traditional South Asian family structure, individuals accept a strict division of labor, subordinating their personal ambitions to the caregiving needs of the family [67]. However, demographic pressures have subjected the informal care system to unprecedented strain, and the ageing trend may be eroding this caregiving orientation.^1^^1^North and Fiske [131] argue that rapid ageing has caused demographic strains in Eastern societies, making ageism and age-based stereotyping more prevalent than in the West. The pandemic exerted an additional stress test, which has thrown a spotlight on the underlying tensions.

The COVID-19 pandemic was a natural experiment enabling researchers to observe how the informal care system stood up during a crisis. As informal caregiving is voluntary and non-monetary, the resulting stresses cannot be measured in dollars. This study conceptualizes the negative impacts as ‘treatments’ requiring emotionally costly adjustments. To evaluate these emotional costs, the study will do three things. First, using survey data from 454 predominantly young, well-educated carers in Bangladesh, India, and Pakistan, it will identify the latent dimensions of pandemic environmental disruption. Second, the study will estimate the effects of these changes (treatments) on two subjective carer responses: change in burden frequency and pandemic burden frequency (from henceforth, ‘burden change’, and ‘pandemic burden’). Third, the study will interpret the analysis outputs, yielding a better qualitative understanding of the stresses experienced by the carers. It will illustrate how globally implemented health policies aimed at limiting contagion created an exogenous shock in the South Asian environment that impacted carers’ ability to perform their duties.

## Methods

This section describes the questionnaire and sample, summarizes the analysis strategy, and presents the variables. The technical details of the analysis are covered in the Supplementary online Appendix.

### CLIC caregiver questionnaire

The data came from *Coping with Loneliness, Isolation, and COVID-19* (CLIC), an international study using a cross-sectional online self-administered survey [68]. The CLIC responses came from over 20,000 adults in over 100 countries, collected between June and November 2020. The embedded CLIC Caregiver study focused on carers of individuals with long-term brain health and physical conditions before and during the pandemic. The survey instrument was developed by an international panel of specialists in loneliness/social isolation, dementia, and other brain health conditions in collaboration with the Alzheimer Society of Ireland (ASI) and Family Carers Ireland. It was developed in English and translated into Urdu and Bengali to increase dissemination.

The effects of the pandemic varied from one country to the next. One goal of the CLIC study was to produce region-specific analyses that explored these unequal impacts, controlling for cultural, religious, and linguistic variations [12]. This study highlights the pandemic’s effects on the well-being of South Asian carers.

### Sample

The participants were informal carers (unpaid spouses, family members, and friends) born and practicing in India, Pakistan, and Bangladesh. They were identified by their affirmative response to ‘Do you provide care and support to a family member or friend with a long-term or life-limiting health problem or disability (including mental health)?’

### Analysis strategy

**Figure 1.**
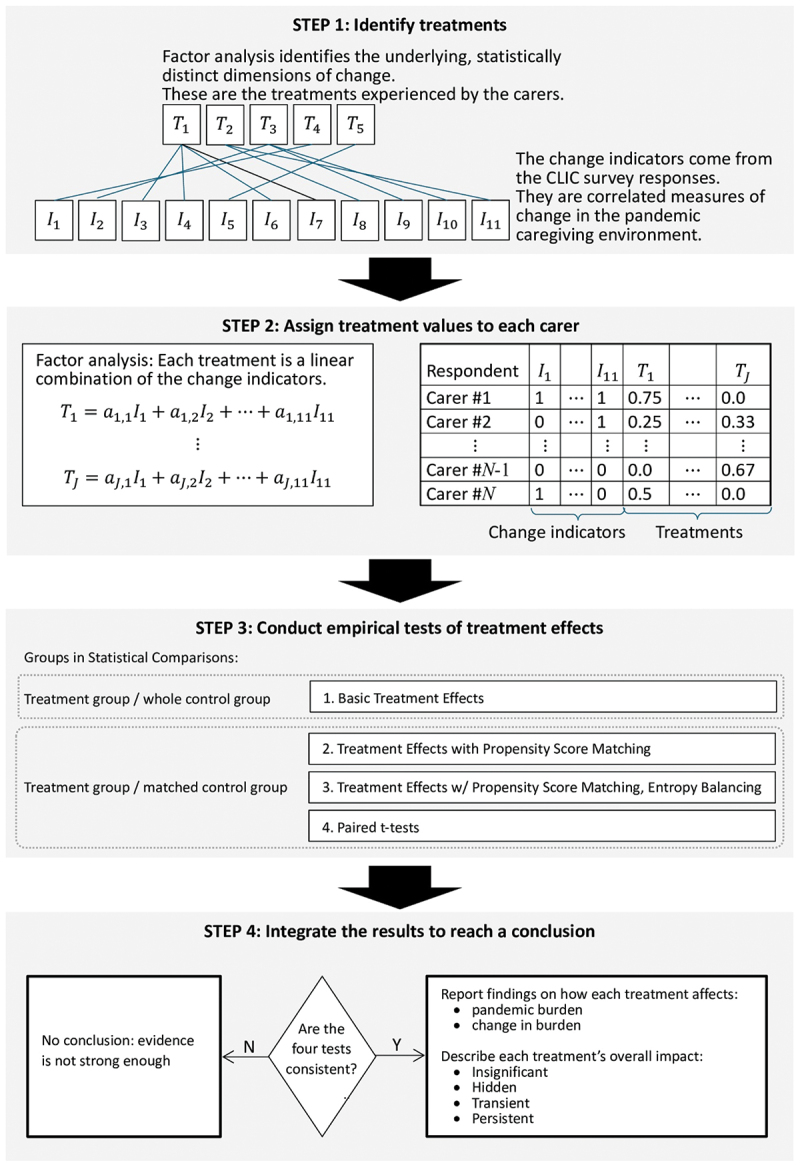
Overview of analysis procedure.

Figure 1 depicts the analysis strategy. An overview of the four steps is presented below. The Supplementary online Appendix provides further technical details.

Step 1 identifies the treatments. Respondents completed an 11-item checklist specifying the ways the pandemic had changed their caregiving environments. These indicators were correlated; therefore, tetrachoric factor analysis was used to distinguish the latent dimensions of environmental change, representing emotional treatments.

Step 2 assigns treatment values to the respondents. Factor analysis expresses the treatments as linear combinations of the change indicators. For each carer, the treatment magnitudes were estimated and appended to the data set.

Step 3 conducts statistical tests to evaluate the impacts of the treatments on the two emotional outcomes, pandemic burden and burden change. In the first three tests, the treatment effects were obtained from the outputs of ordinal logistic regressions. Treatment effects were obtained under three conditions: unmatched control sample, propensity score matching (PSM), and PSM with entropy balancing (EB). The fourth test was a one-sided paired t-test.

**Figure 2.**
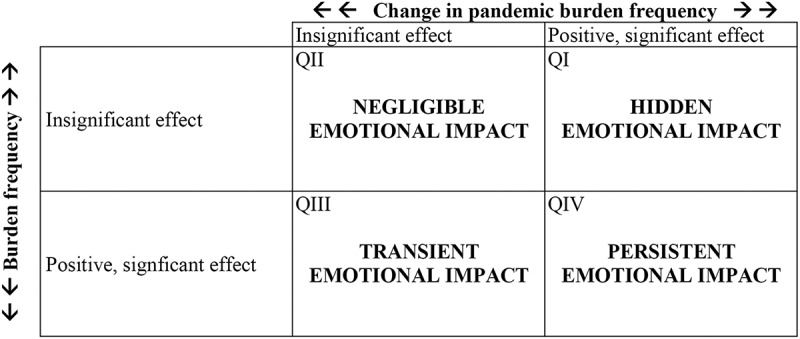
Interpretations of the four possible combinations of treatment effects.

Step 4 integrates the treatment-related findings. When the four tests yielded consistent results, the Figure 2 matrix was used to interpret the two combined treatment effects. The axes represent the impacts on burden change (horizontal) and pandemic burden (vertical). When both effects were insignificant (second quadrant, upper left corner), the treatment had an insignificant impact on the carer’s well-being. A significant effect on burden change only (third quadrant, lower left) indicated a transient impact: burden initially increased but then subsided, either because the change was not lasting or because the carers adjusted to the modified environment, achieving a new equilibrium. A significant effect on pandemic burden only (first quadrant, upper right) suggested a hidden impact: the carer experienced lasting stress without fully recognizing it. When both treatment effects were positive and significant (fourth quadrant, lower right), there was a persistent impact: the carer recognized the burden increase, which had durable psychological consequences lasting into the pandemic.

### Variables

Ordinal logistic specifications were used throughout the analysis to estimate the emotional impact of each treatment on the outcome (burden change or pandemic burden). The variables, including primary stressors, secondary stressors/concordances, and background/context variables [69], are described in detail below.

#### Emotional outcomes

The two dependent variables describe negative emotional outcomes. Pandemic burden was measured by an item from the Zarit Burden Interview (ZBI), ‘How often do you feel burdened in your role during Covid-19?’ [70]; change in burden, by ‘Is it [the burden] the same as usual, more than usual, or less than usual?’

#### Treatments

The changes to the pandemic caregiving environment are conceptualized as emotionally costly treatments. In the CLIC survey, carers completed a checklist specifying changes to their caregiving environments. The treatments, derived from these data via exploratory factor analysis, are described in the Results section below.

#### Primary stressors

The primary stressors describe aspects of the carer/recipient relationship [71,72] that affected care demand. The first is a caregiving venue. Co-residence with the recipient posed risks of overexposure and lower quality of life [73]. Although institutional caregiving can shield carers from overexposure [19], during the pandemic it was associated with restricted access and lack of information/control over the recipient’s well-being.

The second primary stressor is recipient age. During the pandemic, carers assumed additional responsibilities over longer periods [16]. Older recipients, especially those with chronic conditions and disabilities, tended to become increasingly dependent (United Nations ESCAP 2020); child recipients, less so.

The third stressor is the recipient’s medical condition. Carers typically face longer hours, greater physical effort, more job-related complications/conflicts, and higher emotional demands with dementia patients than with patients having only physical conditions [74]. Moreover, a dementia recipient’s functional abilities may decline, while behavioral impairments may increase [75].

#### Secondary stressors/concordances

In the Pearlin et al. [69] framework, secondary stressors capture personal resource availability and role strains that arise when caregiving demands conflict with a carer’s preexisting role(s). This category is extended to include role concordance, a state in which care demands align with the preexisting role, making it easier to meet social expectations.

Caregiving often requires financial sacrifices [76] impacting the whole household. Carers in good financial health can meet their responsibilities without risking family hardship, while those under financial strain, e.g. from pandemic job losses [77], may struggle to fulfill role expectations [78]. This can lead to feelings of shame, inadequacy, and social stigma, as financial insufficiency is a taboo subject in South Asian culture [79] reflecting on an individual’s competence and ability to provide for loved ones.

Caregiving may involve strenuous tasks [80], e.g. positioning or moving the recipient. A carer in robust physical health can more effectively perform such tasks, while one who is in poor health can either neglect them or risk chronic pain and injury [81].

Caregiving can be mentally and emotionally taxing. A carer in sound mental and psychological health is better placed to handle demands without feeling burdened.

Employed carers may face conflicting societal expectations. During the pandemic, when time and energy reserves were reduced, they may have left care demands unmet, causing role strain and guilt. Working carers who find such burdens intolerable may ultimately quit their jobs [82]. By contrast, homemakers may find their responsibilities more compatible with caregiving, making it easier to balance social expectations.

Troy et al. [83] find that highly educated carers face an ‘expectations gap’ when caregiving conflicts with career ambitions [84]. Carers who compare themselves unfavorably to similarly educated peers may experience psychosocial stresses and emotional health issues. Among paid carers, educated females may be seen by employers as less manageable [85]. In interviews with 313 informal carers of dependent older persons, educated respondents were less likely to express positive attitudes towards the elderly [86].

#### Background/context variables

Caregiver burden is a multidimensional process [72] linked to the sociodemographic, economic, and health-specific characteristics of the carer [76,83]. The background/context variables capture the stable characteristics of carers and the caregiving environment.

Gender affects societal expectations concerning caregiving. Traditional South Asian norms place women, particularly daughters and daughters-in-law, at the helm of caregiving responsibilities [67]. Female carers are more exposed to all aspects of caregiving [87], with potentially greater impacts on their careers, health, and well-being [88,89]. The pandemic reportedly worsened this gender imbalance [87].

Age, marital status, and number of children are all control variables associated with the carer’s life cycle status.

Nationality is linked to pandemic changes in the caregiving environment. Although Bangladesh, India, and Pakistan adopted broadly similar COVID-19 containment policies, their enforcement approaches differed [58]. Nationality also captures country-specific demographic trends. In 2020, Pakistan’s age dependency ratio (ADR), the proportion of elderly dependents to working-aged people stood at roughly 7%, *versus* 8% in Bangladesh and 10% in India [90], and Pakistan’s ADR was growing much more slowly. Bangladesh and India have higher human capital indices and per capita GDP. Although these statistics hint that Pakistan may have a stronger caregiving orientation, the relationship appears to be complex. For example, Ng and Indran [91], using corpus-based measures, found that out of 20 countries, Bangladesh, Pakistan, and India ranked 1, 6, and 12 in positive perceptions of carers.

Carer residential setting (e.g. city *versus* village or town) is another background/context variable. In rural areas, medical services were less available to carers [92]. Containment measures were not as vigorously enforced [58].

#### Summary

Table 1 presents the variable definitions.

**Table 1.** Variable definitions.

Variable name/category	Description
Caregiving outcomes	
BFreq	Five-level ordinal response to the question ‘During COVID-19 how often do you feel burdened in your caring role?’
BChange	Three-level ordinal response to the follow-up question, ‘Is this the same as usual, less than usual, or more than usual?’
Environmental change	
$F_{1}$ (Trapped)	Continuous measure reflecting the inescapability of the care setting (i.e. feeling ‘trapped’)
$F_{2}$ (NoInfo)	Continuous measure reflecting the carer’s lack of access to needed medical information
$F_{3}$ (NoConnect)	Continuous measure reflecting the carer’s feeling of losing connection with the recipient and/or others regarding the recipient’s care
$F_{4}$ (NoVisit)	Binary indicator that the carer has lost access to the recipient, e.g. because of a ‘no visitors’ policy at a care facility
$F_{5}$ (PClothing)	Binary indicator that the need to wear protective clothing (e.g. face masks)
Primary stressors	
Venue	Categorical variable identifying one of four possible care venues
RAge	A categorical variable specifying
RPhys	Binary indicator that the care recipient has a physical condition
RDem	Binary indicator that the care recipient has dementia
ROth	Binary indicator that the care recipient has a condition other than physical or dementia
Secondary Stressors	
FHealth	Three-level ordinal measure of carer financial health
PHealth	Three-level ordinal measure of carer physical health
MHealth	Three-level ordinal measure of carer mental health
Educ	Three-level ordinal variable describing the carer’s highest educational qualification
Employment	Categorical variable specifying one of four possible carer employment statuses
Background/context variables	
Male	A binary indicator that the carer is of male gender
Age	An integer variable stating the carer’s age in years
MStatus	A categorical variable associating the carer with one of three marital statuses
NKids	An integer count variable stating how many children the carer has
Country	A categorical variable identifying the carer’s country of orgin as Bangaldesh, India, or Pakistan
ResidSetting	An ordinal variable identifying the carer’s residential setting as city, town, or village

## Results

This section presents the factor analysis interpretations and summarizes the analysis results. The technical details are reported in the Supplementary online Appendix.

### Factor (treatment) interpretations

The loadings (Supplementary online Appendix, Table S-2) suggest that the treatments capture specific aspects of a carer’s pandemic experience. This section interprets the treatments by examining the relevant responses to two open-ended follow-up survey questions, ‘Please describe how this [your ability to give care] has changed [as a result of environmental changes or interruptions]’ and ‘Please describe how you feel about your caring role now?’ For each treatment, responses from carers for whom that treatment was positive but the other treatments were all zero are examined.

#### Treatment 1

##### Trapped

This treatment encodes the carer’s perception that the pandemic caregiving environment was socially confining. There were 43 respondents for whom Trapped was the only positive treatment. Some described the long hours they spent with the recipient and the curtailing of social outlets:

I gave him a lot of time. I was always with him.

Give him too much time.

Unable to meet families and go out whenever need arises, need to be extra precautionary towards the situation.

Others expressed concern about the impact of isolation on the care recipient:

He is living in a flat nearby where we are allowed as we are also health care workers But, other visitors have restricted entry and this is limiting his social interaction greatly.

The stage in dementia has advanced during Covid-19. Going to a daycare before this situation had somehow stabilized the condition.

#### Treatment 2

##### NoInfo

This treatment concerns the reduced availability of information (medical or non-medical) regarding the recipient. Information flows were sometimes impeded by poor communication with hospitals and other medical service providers (e.g. because those providers were overwhelmed by demand and/or discouraged non-essential visits), with the recipient or with others involved in the caregiving arrangements. Because of data limitations, no open-ended responses are available to aid in interpretation. Of the 48 carers for whom NoInfo was positive, most also experienced other treatments. Only four carers had NoInfo as the only positive treatment, none of whom responded to the open-ended questions

#### Treatment 3

##### NoConnect

This treatment captures the carer’s reduced intimacy and connection with the recipient. There were 27 respondents for whom NoConnect was positive and the other treatments were all zero. Their open-ended responses confirmed that the COVID-19 containment measures limited visits and physical proximity to the recipient:

Due to covid 19, I was not able to check his daily routine activities.

Unable to visit her. But thankfully my sister is able to take care

I take utmost precautions to maintain respiratory hygiene and social distancing while communicating with the concerned person now

#### Treatment 4

##### NoVisit

This treatment concerns lack of visitation rights. There were nine respondents for whom NoVisit was the only positive treatment. While three of them did their caregiving at a facility, six (a plurality) did so at the recipient’s residence. The open-ended responses suggest that while some carers regarded the curtailment of in-person visits as mandatory (i.e. in response to lockdown regulations), there may have been other reasons for the reduced contact (e.g. to protect a vulnerable family member from exposure).

*Number of visits becomes lesser and timing becomes short per session*

*Becauz of lockdown cause covid19*

*Due to covid visits have lessen*

#### Treatment 5

##### PClothing

This treatment concerns one environmental change, the need to wear protective clothing. There were eight individuals for whom PClothing was the only positive treatment. Half of them did their caregiving in a facility. The open-ended responses confirm that mask-wearing had an outsized impact on residents.

… need to wear mask all the time, residents will un co-operative towards care some times, challenges in their personal care, maintain social distance also some times cause agitation behavior

Social distancing, using masks – This leading to un co-operative behaviors from the residents.

### Summary of findings

Table 2 summarizes the empirical tests. Two of the five treatments, Trapped and NoConnect, consistently increased both burden change and pandemic burden. Across all four tests, NoVisit increased pandemic burden, but did not influence burden change. Overall, the NoInfo and PClothing effects were either inconsistent or insignificant.

**Table 2.** Summary of the test results.

	Basic (1)	PSM (2)	PSM + EB (3)	Paired t-test (4)	Overall (5)
**Panel A: Change in burden frequency**					
Trapped	+	+	+	+	**+**
NoInfo	+	Insignificant	Insignificant	Insignificant	**Inconsistent**
NoConnect	+	+	+	+	**+**
NoVisit	Insignificant	Insignificant	Insignificant	Insignificant	**Insignificant**
PClothing	Insignificant	Insignificant	Insignificant	Insignificant	**Insignificant**
**Panel B: Pandemic burden frequency**					
Trapped	+	+	+	+	**+**
NoInfo	Insignificant	Insignificant	Insignificant	Insignificant	**Insignificant**
NoConnect	+	+	+	+	**+**
NoVisit	+	+	+	+	**+**
PClothing	+	+	Insignificant	Insignificant	**Inconsistent**

(1) In Columns 1–4, each test was either positive and significant (+) or insignificant.

(2) In Column 5, the overall treatment effect was positive and significant if all four tests were positive and significant, insignificant if all four tests were insignificant, or inconsistent if the tests had more than one outcomes.

## Discussion

This section interprets the empirical results, defines the concept of an Embedded Carer Support Strategy (ECSS), recommends specific caregiving interventions, suggests directions for future research, and summarizes the strengths and limitations of this study.

### Interpretations

The analysis identified three changes in the pandemic caregiving environment that significantly influenced the burden. The first, Trapped, had a persistently negative impact on carer well-being, in keeping with prior research. During the pandemic, cohabiting carers were often confined with their recipients for extended periods, increasing stress. A Hong Kong study of 25 stroke survivor carers found that confinement increased dependency, exhaustion, verbal abuse, and lack of respite [93]. Similarly, a survey of 5,568 Pakistani carers linked the psychological toll of confinement to various demographic factors [94]. These and other findings [95,96] suggest that the containment measures severely disrupted the carers’ social lives.

The NoConnect treatment also had persistent negative impacts on carer well-being. Burden was higher when pandemic conditions suppressed carer–recipient interactions. This finding agrees with previous research showing that pandemic conditions could reduce contact and closeness with the recipient [97], causing emotional difficulties for the carer [98]. Numerous studies report a negative association between carer well-being and care intensity [99,100]. A pre-pandemic study of dementia caregivers in eight European countries [101] found that the burden decreased significantly after the recipient had been institutionalized. However, the NoConnect results imply a more complex interplay, in which the burden rose when the pre-pandemic intensity (the equilibrium level) was ‘forced’ to change (increase or decrease) in undesirable ways. Further research is needed.

The NoVisit treatment affected pandemic burden but not changes in burden. It mainly concerned recipients in facilities or separate households. To prevent contagion, care facilities worldwide took extraordinary measures to protect residents [102]. Carers were often denied visitation rights [103], contravening South Asian cultural expectations that family carers remain involved [104–106]. For dementia patients, Information and Communication Technology (ICT) was an inadequate substitute, reducing emotional closeness with carers [103].^2^^2^Chu, Yee, and Stamatopoulos [132] identified four barriers to ICT effectiveness: lack of technology, remote scheduling issues, poor implementation, and unsuitability for residents.

Carers of recipients in separate households may have perceived a ‘loss of rights’ when visits were discouraged due to mobility restrictions, contagion risks, or community objections [18]. Fear of community spread was significant; in some cases, healthcare workers faced hostility, even violence, from residents [2,18]. In this climate, non-cohabiting carers may have hesitated to visit recipients in other neighborhoods.

The NoVisit treatment was found to have a hidden impact: although it caused higher burden during the pandemic, carers were not conscious of its transitional effect. They may have coped by cognitively disengaging – mentally withdrawing or avoiding thoughts about the loss of visitation [107], a strategy common when stressors feel beyond an individual’s control [108].

Previous research suggests that the NoInfo treatment, reflecting a lack of information about the recipient, should have increased the burden. During the peak contagion, overwhelmed South Asian hospitals prioritized COVID-19 cases, disrupting medical communications with non-COVID patients. Healthcare quality declined, with Raman et al. [59] finding that residents in India’s COVID-19 ‘red zones’ rated medical services as less affordable (OR = 1.917), accessible (OR = 2.458), adequate (OR = 3.015), appropriate (OR = 2.225), and/or continuous (OR = 6.756). Mobility restrictions further limited flows of medical and non-medical information, especially to non-cohabiting carers. However, although the data suggest a link between burden and information, the number of observations treated by NoInfo alone was too small to support a conclusion. Further study is warranted.

The PClothing treatment had an insignificant impact. Open-ended responses suggest that the negative effects mainly impacted carers of residents at facilities, where mask mandates caused behavioral issues. Studies find that PPE could be hot and uncomfortable and weaken carer-recipient closeness [13]. However, South Asian carers may have prioritized other pandemic-related challenges over the PPE requirements.

### Embedded carer support strategies

The COVID-19 pandemic underscored the need of governments to prepare for future public health crises. Part of the preparation is supporting informal carers when their mobility is restricted and/or they cannot access essential services in the usual way. The next section presents specific measures that could work under such conditions. It prioritizes

- Mitigation of the stressors identified in the analysis (social isolation resulting from overexposure to the recipient and disrupted access to the recipient).
- Compatibility with existing public health structures.
- Efficient use of resources.
- Respect for South Asian cultural norms.

The guiding principle, that caregiving policies should align with cultural and socioeconomic constraints, is summarized in the notion of an Embedded Carer Support Strategy (ECSS):

An *Embedded Carer Support Strategy* is a public health intervention designed to reduce the emotional burden of caregivers by integrating sustainable support mechanisms within existing healthcare, social, and community structures.

### Recommended caregiving interventions

The carers’ primary needs are counseling and emotional support. This section describes a counseling program that would operate under a national healthcare system.

Recommendation #1: Counseling will be made accessible via ICT, ensuring that (1) the program functions during lockdowns, and (2) it accommodates carers who are homebound (e.g. because of caregiving duties or conservative family expectations [109]). India’s Tele-MANAS hotline demonstrates that ICT is an effective way to deliver mental health services. Tele-MANAS, established in 2022, now handles 3,500 calls per day, on average [110].

The ICT platform will accommodate heterogeneous client devices. Increasing numbers of South Asians are internet-connected via smartphones and/or laptops [111–113]. These individuals will be able to book video consultations through digital platforms such as India’s government-administered portal, *Ayushman Bharat* Digital Mission (ABDM) [114] or Pakistan’s *Sehat Kahani*, a private tele-health platform that partners with the government [109,115].

Currently, 85% of Indians have access to mobile phones [111], but not necessarily smartphones. The figures for Bangladesh and Pakistan are similar [112,113]. This suggests that an SMS link to counseling services is needed. The link would be activated through a missed call: the user would dial a dedicated number, immediately hang up, and wait for a callback.^3^^3^In India, many apps are activated in this manner [133]. For example, India’s *Aarogya Setu*, a mobile phone-based COVID-19 tracker, included a health self-rating app that could used missed call activation [134]. The counseling center would generate SMS acknowledgement and schedule notification messages. At the appointed time, the counselor would call the client, and the session would begin.

Multimedia (video+audio) group therapy sessions could be hosted with the help of a VoIP-Based platform (e.g. Microsoft Teams) with a dedicated phone number [116].

Recommendation #2: Two types of counselors will be employed. The first are mentors, experienced carers who offer advice on problems concerning caregiving. The second are spiritual advisors, religiously affiliated individuals (e.g. *imams*, *pandits*, or pastors), or other specialists who give guidance/support concerning the illness/loss of a loved one.

South Asian culture requires that counselors be of the same gender as their clients [117]. Most one-on-one counseling will therefore be done by women. Male counselors may play larger roles in group sessions.

As in Tele-MANAS [118], counselors will be able to refer clients to specialists (e.g. psychiatrists). All counselors will be trained/certified in caregiving issues, including social isolation, forced separation from the recipient, and family disputes.

Recommendation #3: A minority of South Asians have neither cell phones nor internet access. During a lockdown, these individuals would lack access to counseling services. The government can promote the availability of low-cost, basic phone models for these carers.

Recommendation #4: The effects of South Asia’s digital divide are concentrated among women [119] and the poor [120], who are less likely to have mobile phones and/or internet access. South Asian governments traditionally aid poor and underserved groups through community health workers (CHWs) [121–126]. These are community residents, predominantly women, who perform outreach to individual households. During the pandemic, CHWs were the front-line providers of healthcare and psychosocial support [127]. The counseling program will employ them to tutor clients in basic digital skills. This program could be modeled on *Internet Saathi* [128], an industry-sponsored program that recruited female volunteers to teach digital literacy skills among women in rural Indian communities.

Recommendation #5: A public health messaging program can be established to raise consciousness concerning the needs of carers. The program will deliver carer-friendly content to viewers. It will feature advice from expert speakers and testimonials (anonymous and non-anonymous) describing real-life challenges faced by carers and their families. The program will present thought-provoking cases involving issues such as sharing caregiving responsibilities within the home, social isolation among carers, ‘no-visit’ situations, and family conflicts. It will be broadcast on TV or radio at regularly scheduled times.

### Future research

This paper suggests that during the pandemic, specific changes in the caregiving environment negatively affected carer emotional well-being. Three treatments stood out for their significant impacts: Trapped, reflecting social isolation and overexposure to the care recipient, and NoConnect, reflecting lack of access/lack of intimacy with the care recipient, and NoVisit, reflecting loss of visitation rights. Further research is needed to examine these environmental impacts in greater detail. In particular, qualitative analyses could reveal the pathways through which they affect carers in different cultural settings.

### Strengths and limitations

A key strength of this study is its methodology. Factor analysis identified five dimensions of pandemic-induced caregiving change, while treatment effects analysis measured their impacts on carer emotional health, revealing the most burdensome changes. Another strength is the focus on South Asian carers, an underrepresented group with distinct socioeconomic caregiving challenges.

The study also has limitations. The retrospective, self-reported survey data may have introduced recall bias and subjective interpretations of burden. The results must be interpreted with caution because of the small sample size in some analyses. The sample is predominantly affluent and well-educated, potentially limiting the generalizability of the findings to the broader South Asian carer population. Although the respondents were predominantly unpaid family carers, some of them may have been paid. Sample weighting was not employed to compensate for the high representation of Pakistani carers (47%) within the sample. Confirmation bias may have influenced the selection of qualitative responses during the interpretation of the identified factors.

## Conclusion

Early in the pandemic, South Asian governments adopted lockdowns, mobility restrictions, and school/work closures, which were standard, globalized responses to COVID-19 contagion [57]. Informal carers underwent emotionally costly adjustments, driven by changes in the local caregiving environment. This study identified five change dimensions (treatments) and estimated the emotional costs of each. Social isolation and separation from the recipient, which caused caregiving intensity to deviate from its pre-pandemic level, were found to increase emotional costs. These findings add to the limited evidence on caregiving in LMICs.

Caregiver burden reflects increasing demands on the carer that lead to emotional strain and self-diminishing values [69]. Our study raises the hope that in future crises, public health interventions will mitigate this private emotional suffering among South Asian carers. Using currently available resources, counseling services could be offered either in person during normal times or remotely (e.g. by phone) during a crisis.

In households throughout the globe, carers provide unpaid assistance and support to vulnerable dependents. They meet the needs of their recipients and assume burdens that might otherwise be handled at public expense. The informal care system will be sustainable only if individual carers remain willing to engage in this work. Carer distress is a public health concern, contributing to burnout and the breakdown of home care arrangements [129]. Informal care is both a public good and an extension of national health systems [24,99]. No society, even the wealthiest, can afford to replace its unpaid carers with hired health workers [130].

## Supplementary Material

Table S2_Factor_loadings.docx

Table S3_Partition_of_the_sample.docx

Table S1_Self_reported impacts on the caregiving environment 2.docx

Table S4_Sample_statistics_CLEAN.docx

Table S5_Empirical_tests.docx

Figure S1_Smoothed Treatment 2 Effect Curves.docx

## Data Availability

The data used in this study are available on request from the corresponding author.

## References

[1] Parkinson A, Matenge S, Desborough J, et al. The impact of COVID-19 on chronic disease management in primary care: lessons for Australia from the international experience. Med J Aust. 2022;216:445–16. doi: 10.5694/mja2.51497 35403236 PMC9114997

[2] Venkata-Subramani M, Roman J. The coronavirus response in India - world’s largest lockdown. Am J Med Sci. 2020;360:742–748. doi: 10.1016/j.amjms.2020.08.002 32892981 PMC7405894

[3] Akhtar H, Afridi M, Akhtar S, et al. Pakistan’s response to COVID-19: overcoming national and international hypes to fight the pandemic. JMIR Public Health Surveill. 2021;7:e28517. doi: 10.2196/28517 33877048 PMC8136406

[4] Hashim A. ‘Smart lockdown’ in Pakistan to target 500 coronavirus hotspots. Al Jazeera [Internet]. 2020 [cited 2025 Feb 13]; Available from: https://www.aljazeera.com/news/2020/6/23/smart-lockdown-in-pakistan-to-target-500-coronavirus-hotspots

[5] Ahmed T, Musarrat P, Kabir ZN. Lessons learned from pandemic response to COVID-19 in Bangladesh: NGO-based emergency response framework for low- and middle-income countries. BMC Health Serv Res. 2023;23:656. doi: 10.1186/s12913-023-09643-w 37340495 PMC10283326

[6] VHA Home HealthCare. Face masks and dementia care [internet]. Toronto: VHA Home HealthCare; 2020 [cited 2025 Feb 5]. Available from: https://www.vha.ca/blog/face-masks-and-dementia-care

[7] Pearl B, Hunter L, Lo K, et al. The enforcement of COVID-19 stay-at-home orders [internet]. Washington, D.C: American Progress; 2020 [cited 2025 Feb 6]. Available from: https://www.americanprogress.org/article/enforcement-covid-19-stay-home-orders/

[8] Zhou X, Song Y, Jiang H, et al. Comparison of public responses to containment measures during the initial outbreak and resurgence of COVID-19 in China: infodemiology study. J Med Internet Res. 2021;23:e26518. doi: 10.2196/26518 33750739 PMC8023317

[9] Agyapon-Ntra K, McSharry PE. A global analysis of the effectiveness of policy responses to COVID-19. Sci Rep. 2023;13:5629. doi: 10.1038/s41598-023-31709-2 37024541 PMC10078072

[10] Varghese GM, John R. COVID-19 in India: moving from containment to mitigation. Indian J Med Res. 2020;151:136–139. doi: 10.4103/ijmr.IJMR_860_20 32317412 PMC7366532

[11] Biswas S. India coronavirus: how Kerala’s COVID ‘success story’ came undone. BBC News [internet]. 2020 [cited 2025 Feb 13]; Available from: https://www.bbc.com/news/world-asia-india-53431672

[12] Grycuk E, Chen Y, Almirall-Sanchez A, et al. Care burden, loneliness, and social isolation in caregivers of people with physical and brain health conditions in English-speaking regions: before and during the COVID-19 pandemic. Int J Geriatr Psychiatry. 2022;37:1–13. doi: 10.1002/gps.5734 PMC9324775 35574817

[13] Markkanen P, Brouillette N, Quinn M, et al. “It changed everything”: the safe home care qualitative study of the COVID-19 pandemic’s impact on home care aides, clients, and managers. BMC Health Serv Res. 2021;21:1055. doi: 10.1186/s12913-021-07076-x 34610836 PMC8491760

[14] Budnick A, Hering C, Eggert S, et al. Informal caregivers during the COVID-19 pandemic perceive additional burden: findings from an ad-hoc survey in Germany. BMC Health Serv Res. 2021;21:353. doi: 10.1186/s12913-021-06359-7 33863337 PMC8050992

[15] Qin VM, Visaria A, Malhotra R. Impact of a COVID-19-related lockdown on the experience of informal caregiving in Singapore. Gerontology. 2024;70:102–114. doi: 10.1159/000534723 37866359 PMC10794967

[16] Hwang Y, Connell LM, Rajpara AR, et al. Impact of COVID-19 on dementia caregivers and factors associated with their anxiety symptoms. Am J Alzheimers Dis Other Dement. 2021;36:15333175211008768. doi: 10.1177/15333175211008768 PMC8573819 33853394

[17] Cross LA, Koren A, Dowling JS, et al. The impact of COVID-19 on family caregivers of individuals with end-stage heart failure. J Hosp Palliat Nurs. 2022;24:249–257. doi: 10.1097/NJH.0000000000000881 35881680 PMC9435954

[18] Mahmud A, Islam MR. Social stigma as a barrier to COVID-19 responses to community well-being in Bangladesh. Int J Community Well-Being. 2021;4:315–321. doi: 10.1007/s42413-020-00071-w PMC7416994 34723103

[19] Hristova C, Ordóñez P, Stripling A, et al. Caregiver burden as impacted by COVID-19: translation of a rapid review to clinical recommendations. Am J Geriatr Psychiatry. 2021;29:S63–S64. doi: 10.1016/j.jagp.2021.01.055

[20] Ranji U, Frederiksen B, Salganicoff A, et al. Women, work, and family during COVID-19: findings from the KFF Women’s health survey [internet]. San Francisco (CA): Kaiser Family Foundation; 2021 [cited 2025 Feb 6]. Available from: https://www.kff.org/mental-health/issue-brief/women-work-and-family-during-covid-19-findings-from-the-kff-womens-health-survey/

[21] Landivar LC, Scarborough WJ, Ruppanner L, et al. Remote schooling and mothers’ employment during the COVID-19 pandemic by race, education, and marital status. RSF Russ Sage Found J Soc Sci. 2023;9:134–158. doi: 10.7758/RSF.2023.9.3.06

[22] García-Vivar C, Fernández-Alcántara M, Falcó-Pegueroles A, et al. Family caregivers’ experiences during the COVID-19 pandemic: a qualitative study. Healthcare (Basel). 2024;12:970. doi: 10.3390/healthcare12100970 38786382 PMC11121002

[23] Perales N, Jiménez B, Caballero D, et al. Informal cares and caregivers in rural elderly: emotional costs in public health policies. In: Research anthology on supporting healthy aging in a digital society. IGI Global; 2022. p. 211–224. doi: 10.4018/978-1-5225-9818-3.ch019

[24] Calvó-Perxas L, Vilalta-Franch J, Litwin H, et al. A longitudinal study on public policy and the health of in-house caregivers in Europe. Health Policy. 2021;125:436–441. doi: 10.1016/j.healthpol.2021.02.001 33602532

[25] Moreland A, Herlihy C, Tynan MA, et al. Timing of state and territorial COVID-19 stay-at-home orders and changes in population movement - United States, March 1-May 31, 2020. MMWR Morb Mortal Wkly Rep. 2020;69:1198–1203. doi: 10.15585/mmwr.mm6935a2 32881851 PMC7470456

[26] Coronavirus: the world in lockdown in maps and charts [internet]. BBC News. 2020 [cited 2025 Feb 9]. Available from: https://www.bbc.com/news/world-52103747

[27] Ballotpedia. State-level mask requirements in response to the coronavirus (COVID-19) pandemic, 2020–2022 [internet]. Ballotpedia. 2022 [cited 2025 Feb 8]. Available from: https://ballotpedia.org/State-level_mask_requirements_in_response_to_the_coronavirus_%28COVID-19%29_pandemic%2C_2020-2022

[28] World Health Organization Regional Office for Europe. Public health and social measures: wearing of masks [internet]. WHO/Europe; [cited 2025 Feb 8]. Available from: https://phsm.euro.who.int/covid-19/masks

[29] Carrera S, Luk N, L V, et al. In the name of COVID-19: an assessment of the Schengen internal border controls and travel restrictions in the EU [internet]. Brussels: European Parliament; 2020 [cited 2025 Feb 9]. Available from: https://www.europarl.europa.eu/RegData/etudes/STUD/2020/659506/IPOL_STU(2020)659506_EN.pdf

[30] Cahill S, Bielsten T, Zarit SH. Developing a framework for the support of informal caregivers: experiences from Sweden, Ireland, and the United States research on aging. 2022;44:647–661. doi: 10.1177/01640275221113356 35794800

[31] Hart PS, Chinn S, Soroka S. Politicization and polarization in COVID-19 news coverage. Sci Commun. 2020;42:679–697. doi: 10.1177/1075547020950735 38602988 PMC7447862

[32] Howard J. Face mask guidance has changed. Here’s why [internet]. CNN; 2020 [cited 2025 Feb 9]. Available from: https://edition.cnn.com/2020/06/25/health/face-mask-guidance-covid-19/index.html

[33] Bariola N, Collins C. The gendered politics of pandemic relief: labor and family policies in Denmark, Germany, and the United States during COVID-19. Am Behav Sci. 2021;65:1671–1697. doi: 10.1177/00027642211003140 38603053 PMC7992092

[34] European Commission. Communication from the commission: COVID-19 guidelines for border management measures to protect health and ensure the availability of goods and essential services [internet]. Off J Eur union. 2020 [cited 2025 Feb 9]. Available from: https://eur-lex.europa.eu/legal-content/EN/TXT/PDF/?uri=CELEX:52020XC0330(02)

[35] Dezan Shira & Associates. China’s travel restrictions due to COVID-19: an explainer [Internet]. China briefing. 2020 [cited 2025 Feb 8]. Available from: https://www.china-briefing.com/news/chinas-travel-restrictions-due-to-covid-19-an-explainer/

[36] Morgan Lewis & Bockius LLP. COVID-19 in Japan: travel ban now includes United States, other major trading partners [internet]. Morgan Lewis. 2020 [cited 2025 Feb 8]. Available from: https://www.morganlewis.com/pubs/2020/04/covid-19-in-japan-travel-ban-now-includes-united-states-other-major-trading-partners-cv-lf

[37] Ministry of Foreign Affairs, Republic of China (Taiwan). Taiwan to bar foreign nationals from entering the country starting March 19 in response to the continued spread of COVID-19 [internet]. Taipei (Taiwan): Ministry of Foreign Affairs; 2020 [cited 2025 Feb 9]. Available from: https://www.mofa.gov.tw/en/News_Content.aspx?n=1EADDCFD4C6EC567&s=1816E365AF525FDD

[38] Center for Strategic and International Studies (CSIS). Timeline: South Korea’s response to COVID-19 [internet]. Washington, DC: CSIS; [cited 2025 Feb 9]. Available from: https://www.csis.org/analysis/timeline-south-koreas-response-covid-19

[39] Kim D, Liu M, Rodriguez D, et al. Digital innovations for pandemic response in Asia: lessons for Canada. Asia Pac Found Canada. 2022;2. Available from: https://www.asiapacific.ca/sites/default/files/inline-files/PHAC-Paper2_MARCH.pdf

[40] Haerpfer C, Inglehart R, Moreno A, et al. World values survey wave 7 (2017–2022) cross-national data-set [internet]. Version 4.0.0. Madrid (Spain) & Vienna (Austria): World Values Survey Association; 2022 [cited 2025 Feb 9]. doi: 10.14281/18241.18

[41] U.S. Department of the Treasury. Paycheck protection program [internet]. Washington, DC: U.S. Department of the Treasury; 2021 [cited 2025 Feb 9]. Available from: https://home.treasury.gov/policy-issues/coronavirus/assistance-for-small-businesses/paycheck-protection-program

[42] Public Citizen. Corporations that received billions during the pandemic laid off thousands of workers and gave CEOs millions [internet]. Washington, DC: Public Citizen; 2021 [cited 2025 Feb 9]. Available from: https://www.citizen.org/news/corporations-that-received-billions-during-the-pandemic-laid-off-thousands-of-workers-and-gave-ceos-millions/

[43] Institute for Government. Coronavirus: how countries supported wages during the pandemic [internet]. London: Institute for Government; 2021 [cited 2025 Feb 9]. Available from: https://www.instituteforgovernment.org.uk/article/explainer/coronavirus-how-countries-supported-wages-during-pandemic

[44] BBC News. Japan unveils record stimulus to boost economy [internet]. BBC News. 2020 [cited 2025 Feb 9]; Available from: https://www.bbc.com/news/business-55226200

[45] National Credit Union Administration. Summary of the coronavirus Aid, relief, and economic security (CARES) act [internet]. NCUA; 2020 [cited 2025 Feb 9]. Available from: https://ncua.gov/regulation-supervision/letters-credit-unions-other-guidance/summary-coronavirus-aid-relief-and-economic-security-cares-act

[46] European Central Bank. Short-time work schemes and their effects on wages and disposable income. ECB Economic Bulletin [Internet]. 2020 [cited 2025 Feb 9]; Available from: https://www.ecb.europa.eu/press/economic-bulletin/focus/2020/html/ecb.ebbox202004_06~6b0e718192.en.html

[47] El País. Spain to approve guaranteed minimum income scheme for vulnerable families [internet]. El País. 2020 [cited 2025 Feb 9]. Available from: https://english.elpais.com/spanish_news/2020-05-29/spain-to-approve-guaranteed-minimum-income-scheme-for-vulnerable-families.html

[48] Kubota S, Onishi K, Toyama Y. Consumption responses to COVID-19 payments: evidence from a natural experiment and bank account data. J Econ Behav Organ. 2021;188:1–17. doi: 10.1016/j.jebo.2021.05.006 Epub 2021 May 14. 34566217 PMC8450669

[49] Center for Infectious Disease Research and Policy. COVID-19 to cost U.S. hospitals $200 billion through June [internet]. Minneapolis (MN): CIDRAP; 2020 [cited 2025 Feb 9]. Available from: https://www.cidrap.umn.edu/covid-19/covid-19-cost-us-hospitals-200-billion-through-june

[50] European Commission. EU solidarity fund: COVID-19 response [Internet]. Brussels: European Commission; 2020 [cited 2025 Feb 9]. Available from: https://ec.europa.eu/regional_policy/funding/solidarity-fund/covid-19_en

[51] BBC News. China’s Wuhan builds hospital in 10 days to fight coronavirus [internet]. BBC News. 2020 Feb 2 [cited 2025 Feb 9]. Available from: https://www.bbc.com/news/av/world-asia-china-51348297

[52] Centers for Disease Control and Prevention. Traveler-based SARS-CoV-2 genomic surveillance program [internet]. Atlanta (GA): CDC; 2021 [cited 2025 Feb 9]. Available from: https://www.cdc.gov/advanced-molecular-detection/php/success-stories/airport-genomic-surveillance.html

[53] U.S. Department of Transportation. Runway to recovery: the United States framework for airlines and airports to mitigate the public health risks of coronavirus. Internet. Washington, DC: U.S. Department of Transportation; 2020 Dec [cited 2025 Feb 9]. Available from: https://www.transportation.gov/sites/dot.gov/files/2020-12/Runway_to_Recovery_1.1_DEC2020_Final.pdf

[54] European Commission. Special report on the impact of COVID-19 on air navigation service provision in Europe and the US [internet]. Brussels: European Commission; 2021 Dec 8 [cited 2025 Feb 9]. Available from: https://transport.ec.europa.eu/news-events/news/special-report-impact-covid-19-air-navigation-service-provision-europe-and-us-2021-12-08_en

[55] Reuters. Cathay pacific completes buyback of warrants worth HK$1.53 bln from Hong Kong government [internet]. Reuters. 2024 [cited 2025 Feb 9]. Available from: https://www.reuters.com/business/aerospace-defense/cathay-pacific-completes-buyback-warrants-worth-hk153-bln-hong-kong-government-2024-09-13

[56] World Health Organization. 2019 novel coronavirus (2019-nCoV): strategic preparedness and response plan [internet]. Geneva: World Health Organization; 2020 [cited 2024 May 16]. Available from: https://www.who.int/docs/default-source/coronaviruse/srp-04022020.pdf

[57] Pincombe M, Reese V, Dolan CB. The effectiveness of national-level containment and closure policies across income levels during the COVID-19 pandemic: an analysis of 113 countries. Health Policy Plan. 2021;36:1152–1162. doi: 10.1093/heapol/czab054 PMID: 33942081; PMCID: PMC8135717. 33942081 PMC8135717

[58] Adeel A, Zhirnov A. COVID-19 response in India, Pakistan, and Bangladesh: shared history, different processes. In: Shvetsova O, editor. Government responses to the COVID-19 pandemic. Palgrave Macmillan; 2023. p. 75–106. doi: 10.1007/978-3-031-30844-4_4

[59] Raman R, Rajalakshmi R, Surya J, et al. Impact on health and provision of healthcare services during the COVID-19 lockdown in India: a multicentre cross-sectional study. BMJ Open. 2021;11:e043590. doi: 10.1136/bmjopen-2020-043590 PMC7817386 33468529

[60] Faruqui N, Raman VR, Shiv J, et al. Informal collectives and access to healthcare during India’s COVID-19 second wave crisis. BMJ Glob Health. 2021;6:e006731. doi: 10.1136/bmjgh-2021-006731 PMC8282415 34257140

[61] Abraham DA, Vijayakumar TM, Rajanandh MG. Challenges of non-COVID-19 patients with chronic illness during the pandemic. J Res Pharm Pract. 2020;9:155–157. doi: 10.4103/jrpp.JRPP_20_64 33489985 PMC7808183

[62] Feroz AS, Pradhan NA, Ahmed ZH, et al. Perceptions and experiences of healthcare providers during COVID-19 pandemic in Karachi, Pakistan: an exploratory qualitative study. BMJ Open. 2021;11:e048984. doi: 10.1136/bmjopen-2021-048984 PMC8338319 34344683

[63] Vaitheswaran S, Lakshminarayanan M, Ramanujam V, et al. Experiences and needs of caregivers of persons with dementia in India during the COVID-19 pandemic—a qualitative study. Am J Geriatr Psychiatry. 2020;28:1185–1194. doi: 10.1016/j.jagp.2020.06.026 32736918 PMC7340037

[64] Nisha SI, Bakul F. Parents/Primary caregivers’ perspectives on the well-being, and home-based learning of children with neurodevelopmental disorders during COVID-19 in Bangladesh. Int J Dev Disabil. 2024;70:594–603. doi: 10.1080/20473869.2022.2121140 38983499 PMC11229763

[65] Giebel C, Hanna K, Cannon J, et al. A qualitative 5-country comparison of the perceived impacts of COVID-19 on people living with dementia and unpaid carers. BMC Geriatr. 2022;22:112. doi: 10.1186/s12877-022-02821-1 35148712 PMC8840054

[66] Salve S, Raven J, Das P, et al. Community health workers and COVID-19: cross-country evidence on their roles, experiences, challenges and adaptive strategies. PLOS Glob Public Health. 2023;3:e0001447. doi: 10.1371/journal.pgph.0001447 36962877 PMC10022071

[67] Uddin M. Addressing work-life balance challenges of working women during COVID-19 in Bangladesh. Int Soc Sci J. 2021;71:7–20. doi: 10.1111/issj.12267 PMC8251227 34230685

[68] Chen YS, Beber BC, Higgins D, et al. COVID‐19‐related loneliness and social isolation in caregivers of people with brain health challenges: the CLIC‐Caregiver global survey. Alzheimers Dement. 2021;17:e054161. doi: 10.1002/alz.054161

[69] Pearlin LI, Mullan JT, Semple SJ, et al. Caregiving and the stress process: an overview of concepts and their measures. Gerontologist. 1990;30:583–594. doi: 10.1093/geront/30.5.583 2276631

[70] Siegert RJ, Jackson D, Tennant A, et al. Factor analysis and Rasch analysis of the Zarit Burden Interview for acquired brain injury carer research. J Rehabil Med. 2010;42:302–309. doi: 10.2340/16501977-0511 20461331

[71] Trivedi R, Lorenz KA. Supporting Indian and other South asians facing COVID-19 and other serious illnesses. J Pain Symptom Manag. 2021;62:e1–3. doi: 10.1016/j.jpainsymman.2021.06.024 PMC8479549 34271145

[72] Park M, Sung M, Kim SK, et al. Multidimensional determinants of family caregiver burden in Alzheimer’s disease. Int Psychogeriatr. 2015;27:1355–1364. doi: 10.1017/S1041610215000460 25853717

[73] Metzelthin SF, Verbakel E, Veenstra MY, et al. Positive and negative outcomes of informal caregiving at home and in institutionalised long-term care: a cross-sectional study. BMC Geriatr. 2017;17:1–10. doi: 10.1186/s12877-017-0620-3 29017453 PMC5635563

[74] Kim Y, Schulz R. Family caregivers’ strains: comparative analysis of cancer caregiving with dementia, diabetes, and frail elderly caregiving. J Aging Health. 2008;20:483–503. doi: 10.1177/0898264308317533 18420838

[75] van den Kieboom R, Snaphaan L, Mark R, et al. The trajectory of caregiver burden and risk factors in dementia progression: a systematic review. J Alzheimers Dis. 2020;77:1107–1115. doi: 10.3233/JAD-200647 32804093 PMC7683084

[76] Lai DW. Effect of financial costs on caregiving burden of family caregivers of older adults. Sage Open. 2012;2:2158244012470467. doi: 10.1177/2158244012470467

[77] Rasul G, Nepal AK, Hussain A, et al. Socio-economic implications of COVID-19 pandemic in South Asia: emerging risks and growing challenges. Front Sociol. 2021;6:629693. doi: 10.3389/fsoc.2021.629693 33869579 PMC8022444

[78] Nijboer C, Triemstra M, Tempelaar R, et al. Determinants of caregiving experiences and mental health of partners of cancer patients. Cancer. 1999;86:577–588. doi: 10.1002/(SICI)1097-0142(19990815)86:4<577::AID-CNCR6>3.0.CO;2-S 10440685

[79] Sahu D. The money taboo in India [internet]. Times of India. 2022 [cited 2022 Dec 10]. Available from: https://timesofindia.indiatimes.com/readersblog/intelligibility/the-money-taboo-in-india-45390/

[80] Geere JL, Gona J, Omondi FO, et al. Caring for children with physical disability in Kenya: potential links between caregiving and carers’ physical health. Child Care Health Dev. 2013;39:381–392. doi: 10.1111/j.1365-2214.2012.01398.x 22823515 PMC3654176

[81] Tong HC, Haig AJ, Nelson VS, et al. Low back pain in adult female caregivers of children with physical disabilities. Arch Pediatr Adolesc Med. 2003;157:1128–1133. doi: 10.1001/archpedi.157.11.1128 14609905

[82] Mohammed SF, Ghaith RF. Relationship between burden, psychological well-being, and social support among caregivers of mentally ill patients. Egypt Nurs J. 2018;15:268–280. doi: 10.4103/ENJ.ENJ_17_18

[83] Troy C, Tjin A, Jj PC, et al. Personal determinants of burden among Indonesian female caregivers of older adults in Taiwan. J Appl Gerontol. 2022;41:217–226. doi: 10.1177/0733464820972888 33238777

[84] Dunlavy AC, Garcy AM, Rostila M. Educational mismatch and health status among foreign-born workers in Sweden. Soc Sci Med. 2016;154:36–44. doi: 10.1016/j.socscimed.2016.02.018 26943012

[85] Lan PC. Maid or madam? Filipina migrant workers and the continuity of domestic labor. Gend Soc. 2003;17:187–208. doi: 10.1177/0891243202250730

[86] Luchesi BM, da Silva Alexandre T, de Oliveira NA, et al. Factors associated with attitudes toward the elderly in a sample of elderly caregivers. Int Psychogeriatr. 2016;28:2079–2089. doi: 10.1017/S1041610216001538 27645519

[87] Chauhan P. Gendering COVID-19: impact of the pandemic on women’s burden of unpaid work in India. Gend Issues. 2021;38:395–419. doi: 10.1007/s12147-020-09269-w PMC7585488 33132690

[88] Gupta R, Pillai VK. Analysis of caregiver burden in South Asian families in the Dallas-Fort Worth metropolitan area: insights for social practice. J Appl Sociol. 2005;2:35–54. doi: 10.1177/19367244052200203

[89] Ugargol AP, Bailey A. Family caregiving for older adults: gendered roles and caregiver burden in emigrant households of Kerala, India. Asian Popul Stud. 2018;14:194–210. doi: 10.1080/17441730.2017.1412593

[90] World Bank. Age dependency ratio (% of working-age population) [internet]. Washington, DC: World Bank; 2021 [cited 2024 Oct 1]. Available from: https://data.worldbank.org/indicator/SP.POP.DPND/

[91] Ng R, Indran N, Khan HTA. Societal perceptions of caregivers linked to culture across 20 countries: evidence from a 10-billion-word database. PLOS ONE. 2021;16:e0251161. doi: 10.1371/journal.pone.0251161 34197470 PMC8248619

[92] Brinda EM, Rajkumar AP, Enemark U, et al. Cost and burden of informal caregiving of dependent older people in a rural Indian community. BMC Health Serv Res. 2014;14:207. doi: 10.1186/1472-6963-14-207 24886051 PMC4022434

[93] Lee JJ, Tsang WN, Yang SC, et al. Qualitative study of Chinese stroke caregivers’ caregiving experience during the COVID-19 pandemic. Stroke. 2021;52:1407–1414. doi: 10.1161/STROKEAHA.120.032250 33588588

[94] Abbas S, Nasir JA. Estimating the social burden of COVID-19 among caregivers of COVID-19 patients in Punjab, Pakistan. Sci Rep. 2024;14:29614. doi: 10.1038/s41598-024-74613-z 39609500 PMC11604926

[95] Cagnin A, Di Lorenzo R, Marra C, et al. Behavioral and psychological effects of coronavirus disease-19 quarantine in patients with dementia. Front Psychiatry. 2020;11:578015. doi: 10.3389/fpsyt.2020.578015 33033486 PMC7509598

[96] Panerai S, Prestianni G, Musso S, et al. The impact of COVID-19 confinement on the neurobehavioral manifestations of people with major neurocognitive disorder and on the level of burden of their caregivers. Life Span Disabil. 2020;23:303–320. Available from: https://lifespanjournal.oasi.en.it/Client/rivista/ENG102_Full%20Issue_Life%20Span%20and%20Disability_XXIII-2_2020.pdf#page=139

[97] Rosalynn Carter Institute for Caregivers. Caregivers in crisis: a report on the impact of the COVID-19 pandemic on family caregivers [internet]. Americus (GA): Rosalynn Carter Institute; 2020 [cited 2025 Feb 9]. Available from: https://rosalynncarter.org/wp-content/uploads/2020/10/Caregivers-in-Crisis-Report-October-2020-10-22-20.pdf

[98] Leggett A, Koo HJ, Park B, et al. The changing tides of caregiving during the COVID-19 pandemic: how decreasing and increasing care provision relates to caregiver well-being. J Gerontol Series B. 2022;77:S86–S97. doi: 10.1093/geronb/gbac002 PMC9122649 35032387

[99] Cohen SA, Kunicki ZJ, Drohan MM, et al. Exploring changes in caregiver burden and caregiving intensity due to COVID-19. Gerontol Geriatr Med. 2021;7:2333721421999279. doi: 10.1177/2333721421999279 33718523 PMC7919204

[100] Kolodziej IWK, Coe NB, Van Houtven CH, et al. The impact of care intensity and work on the mental health of family caregivers: losses and gains. J Gerontol Series B. 2022;77:S98–S111. doi: 10.1093/geronb/gbac031 PMC9122646 35191980

[101] Bleijlevens MH, Stolt M, Stephan A, et al. Changes in caregiver burden and health-related quality of life of informal caregivers of older people with dementia: evidence from the European RightTimePlaceCare prospective cohort study. J Adv Nurs. 2015;71:1378–1391. doi: 10.1111/jan.12561 Epub 2014 Nov 17. PMID: 25403434. 25403434

[102] Wang D, Schmillen AD, Glinskaya EE, et al. The elderly care response to COVID-19: a review of international measures to protect the elderly living in residential facilities and implications for Malaysia. Washington, DC: World Bank Group; 2020. Available from: http://documents.worldbank.org/curated/en/770271591601451349

[103] Mitchell LL, Albers EA, Birkeland RW, et al. Caring for a relative with dementia in long-term care during COVID-19. J Am Med Dir Assoc. 2022;23:428–33.e1. doi: 10.1016/j.jamda.2021.11.026 34929196 PMC8677585

[104] Chadda R. Influence of the new mental health legislation in India. BJPsych Int. 2020;17:20–22. doi: 10.1192/bji.2019.18 34287412 PMC8277537

[105] Chakrabarti S. Cultural aspects of caregiver burden in psychiatric disorders. World J Psychiatry. 2013;3:85–92. doi: 10.5498/wjp.v3.i4.85

[106] Park J, Pardosi J, Islam M, et al. What does family involvement in care provision look like across hospital settings in Bangladesh, Indonesia, and South Korea? BMC Health Serv Res. 2022;22:922. doi: 10.1186/s12913-022-08278-7 35841023 PMC9286761

[107] Carver CS, Scheier MF, Weintraub JK. Assessing coping strategies: a theoretically based approach. J Pers Soc Psychol. 1989;56:267–283. doi: 10.1037/0022-3514.56.2.267 PMID: 2926629. 2926629

[108] Dijkstra MTM, Homan AC. Engaging in rather than disengaging from stress: effective coping and perceived control. Front Psychol. 2016;7:1415. doi: 10.3389/fpsyg.2016.01415 PMID: 27679573; PMCID: PMC5026254. 27708603 PMC5030286

[109] Khan S. Sehat Kahani is showing Pakistan that digital health services can change lives – for both patients and doctors. Gavi, the vaccine alliance. 2023. Available from: https://www.gavi.org/vaccineswork/sehat-kahani-showing-pakistan-digital-health-services-can-change-lives-both

[110] Press Information Bureau. Government of India. Tele-MANAS: national tele mental health programme. Press information bureau. 2022. Available from: https://pib.gov.in/PressReleaseIframePage.aspx?PRID=2022057

[111] Waghmare A. Access to phones and the internet. Data for India. 2024 [updated 2025 Jan 9; cited 2025 Feb 12]. Available from: https://www.dataforindia.com/comm-tech/

[112] Hasan M. Women still lag in mobile ownership, internet adoption. The daily star. 2024 May 19 [cited 2025 Feb 12]. Available from: https://www.thedailystar.net/business/news/women-still-lag-mobile-ownership-internet-adoption-3613001

[113] Pakistan Telecommunication Authority. Telecom indicators [internet]. Islamabad (Pakistan): Pakistan Telecommunication Authority; [cited 2025 Feb 12]. Available from: https://www.pta.gov.pk/category/telecom-indicators

[114] Sharma RS, Rohatgi A, Jain S, et al. The ayushman bharat digital mission (ABDM): making of India’s digital health story. CSIT. 2023;11:3–9. doi: 10.1007/s40012-023-00375-0 PMCID: PMC10064942.

[115] Sehat K. Sehat kahani – telemedicine for a healthier Pakistan. [cited 2025 Feb 11]. Available from: https://sehatkahani.com/

[116] Microsoft. Plan audio conferencing for. Microsoft teams meetings [internet]. Redmond (WA): Microsoft; 2025 [cited 2025 Feb 14]. Available from: https://learn.microsoft.com/nb-no/microsoftteams/audio-conferencing-in-office-365

[117] Tjin A, Goodwin A, Troy C, et al. Balancing duty, stigma, and caregiving needs of people with neurodevelopmental or neurocognitive disorders during a public health emergency in South Asia: a qualitative study of carer experiences. Int J Geriatr Psychiatry. 2024;39:e70010. doi: 10.1002/gps.70010 39558492

[118] Press Information Bureau, Government of India. National tele-mental health program tele-MANAS launched on world mental health day [internet]. New Delhi: Press Information Bureau; 2022 [cited 2025 Feb 13]. Available from: https://pib.gov.in/PressReleasePage.aspx?PRID=1866498

[119] GSMA. The mobile gender gap in South Asia is now widening [internet]. London: GSMA; [cited 2025 Feb 14]. Available from: https://www.gsma.com/solutions-and-impact/connectivity-for-good/mobile-for-development/blog/the-mobile-gender-gap-in-south-asia-is-now-widening/

[120] Kamat R. No country for old men: digital development and accessibility in the global south [internet]. Global souths hub. 2024 [cited 2025 Feb 14]. Available from: https://globalsouth.org/2024/10/no-country-for-old-men-digital-development-and-accessibility-in-the-global-south/

[121] Ministry of health and family welfare, government of India. Guidelines for ASHA and Mahila arogya samiti in the Urban context [internet]. National urban health mission. 2014 [cited 2025 Feb 11]. Available from: https://nhm.gov.in/images/pdf/NUHM/Guidelines_for_Asha_and_MAS_in_Urban_Context.pdf

[122] Ministry of health and family welfare, government of India. Guidelines on accredited social health activists (ASHA) [internet]. National Rural Health Mission. [year unknown] [cited 2025 Feb 11]. Available from: https://nhm.gov.in/images/pdf/communitisation/task-group-reports/guidelines-on-asha.pdf

[123] World Health Organization. India’s ASHA workers win global health leaders award [internet]. 2022 [cited 2025 Feb 11]. Available from: https://www.who.int/india/india-asha-workers

[124] World Health Organization. Bangladesh: community health workers at the heart of a stronger health system and the fight against COVID-19 [internet]. 2021 [cited 2025 Feb 11]. Available from: https://www.who.int/news-room/feature-stories/detail/bangladesh-community-health-workers-at-the-heart-of-a-stronger-health-system-and-the-fight-against-covid-19

[125] Women Deliver. The Pakistani lady health worker program: providing care to underserved populations [internet]. (NY): Women Deliver; [cited 2025 Feb 14]. Available from: https://womendeliver.org/pakistani-lady-health-worker-program-providing-care-underserved-populations/

[126] Afsana K, Alam MA, Chen N, et al. Community health workers in Bangladesh. Exemplars glob health. 2020. Available from: https://www.exemplars.health/topics/community-health-workers/bangladesh

[127] Roy S, Kennedy S, Hossain S, et al. Examining roles, support, and experiences of community health workers in Bangladesh during COVID-19: a mixed methods study. Glob Health Sci Pract. 2022;10:e2200102. doi: 10.9745/GHSP-D-22-00102 PMC9426994 36041841

[128] Bathija M. Internet saathi: improving digital literacy among women [internet]. Forbes India; 2018 [cited 2025 Feb 14]. Available from: https://www.forbesindia.com/article/future-of-work/internet-saathi-improving-digital-literacy-among-women/50951/1

[129] Butler SS, Turner W, Kaye LW, et al. Depression and caregiver burden among rural elder caregivers. J Gerontol Soc Work. 2005;46:47–63. doi: 10.1300/J083v46n01_04 16338884

[130] Shaji KS, Reddy MS. Caregiving: a public health priority. Indian J Psychol Med. 2012;34:303–305. doi: 10.4103/0253-7176.108191 23723535 PMC3662124

[131] North MS, Fiske ST. Modern attitudes toward older adults in the aging world: a cross-cultural meta-analysis. Psychol Bull. 2015;141:993–1021. doi: 10.1037/a0039469 26191955

[132] Chu CH, Yee A, Stamatopoulos V. Poor and lost connections: essential family caregivers’ experiences using technology with family living in long-term care homes during COVID-19. J Appl Gerontol. 2022;41:1547–1556. doi: 10.1177/07334648221081850 35416076 PMC9014337

[133] Anand S. 5 things you can get in India with a missed call. The wall street journal. 2016. Available from: https://blogs.wsj.com/briefly/2016/05/13/5-things-five-things-you-can-get-in-india-by-using-a-missed-call/

[134] National Informatics Centre. Aarogya Setu App [Internet]. New Delhi (India). Informatics. 2020;29:8–10. Available from: https://informatics.nic.in/uploads/pdfs/34072fe7_2_aarogya_setu_app.pdf

