# Supplementary material for: The emotional cost of containment: a cross-sectional analysis of treatment effects among informal carers in South Asia during the COVID-19 pandemic
Source: Glob Health Action. 2025 Jun 3;18(1):2504227. doi: 10.1080/16549716.2025.2504227 (PMC12135087; doi:10.1080/16549716.2025.2504227)
Supplement: Table S2_Factor_loadings.docx [file ZGHA_A_2504227_SM8358.docx]

Table S-2: Factor loadings obtained from exploratory factor analysis

| Indicator | Variable | Factor 1 | Factor 2 | Factor 3 | Factor 4 | Factor 5 |
| --- | --- | --- | --- | --- | --- | --- |
| $I_{1}$ | $H$ | 0.1418 | **0.7302** | 0.0641 | -0.1156 | -0.0517 |
| $I_{2}$ | $I$ | 0.1115 | **0.8433** | 0.1948 | 0.1362 | 0.1646 |
| $I_{3}$ | $S$ | **0.5759** | 0.3634 | 0.0265 | 0.4556 | 0.0915 |
| $I_{4}$ | $R$ | **0.5585** | 0.3274 | 0.1982 | 0.1744 | 0.2962 |
| $I_{5}$ | $V$ | -0.2167 | 0.0190 | 0.1165 | **0.6802** | -0.0074 |
| $I_{6}$ | $F$ | 0.0581 | 0.0455 | **0.5747** | 0.1414 | 0.0823 |
| $I_{7}$ | $W$ | 0.0951 | 0.4705 | **0.6328** | 0.0258 | -0.2333 |
| $I_{8}$ | $G$ | 0.1010 | 0.2619 | **0.5494** | 0.0800 | 0.2324 |
| $I_{9}$ | $E$ | **0.6407** | 0.0637 | 0.0984 | -0.1879 | 0.1431 |
| $I_{10}$ | $C$ | **0.8933** | 0.0716 | 0.0352 | -0.1053 | 0.1212 |
| $I_{11}$ | $P$ | 0.3861 | 0.1132 | -0.0326 | 0.0049 | **0.6919** |

Note: Factor analysis was carried out on a tetrachoric correlation matrix obtained from the 11 change indicators. The factors were extracted through principle components analysis. Rotation was carried out using the orthogonal varimax approach.
