# Supplementary material for: The emotional cost of containment: a cross-sectional analysis of treatment effects among informal carers in South Asia during the COVID-19 pandemic
Source: Glob Health Action. 2025 Jun 3;18(1):2504227. doi: 10.1080/16549716.2025.2504227 (PMC12135087; doi:10.1080/16549716.2025.2504227)
Supplement: Table S3_Partition_of_the_sample.docx [file ZGHA_A_2504227_SM8357.docx]

Table S-3: Partition of the sample by factor (treatment) values

| Subsample | Condition | $N$ |
| --- | --- | --- |
| Control group | $T_{j}=0$, $j=1,\cdots,5$ | 233 |
| Subsample 1 | $T_{1}>0$, $T_{2}=T_{3}=T_{4}=T_{5}=0$ | 43 |
| Subsample 2 | $T_{2}>0$, $T_{1}=T_{3}=T_{4}=T_{5}=0$ | 4 |
| Subsample 3 | $T_{3}>0$, $T_{1}=T_{2}=T_{4}=T_{5}=0$ | 27 |
| Subsample 4 | $T_{4}>0$, $T_{1}=T_{2}=T_{3}=T_{5}=0$ | 13 |
| Subsample 5 | $T_{5}>0$, $T_{1}=T_{2}=T_{3}=T_{4}=0$ | 8 |
| Mixed factors | $T_{j}>0$ for more than one $j$, $j=1,\cdots,5$ | 126 |
| **Total sample size** | **All values of** $\boldsymbol{T}_{\boldsymbol{j}}$ | **454** |

Note: The treatment analyses were based on separate regressions pairing Subsamples 1, …, 5 with the Control group. These regressions excluded the 126 observations for which more than one factor was nonzero.
