# Supplementary material for: The emotional cost of containment: a cross-sectional analysis of treatment effects among informal carers in South Asia during the COVID-19 pandemic
Source: Glob Health Action. 2025 Jun 3;18(1):2504227. doi: 10.1080/16549716.2025.2504227 (PMC12135087; doi:10.1080/16549716.2025.2504227)
Supplement: Table S1_Self_reported impacts on the caregiving environment 2.docx [file ZGHA_A_2504227_SM8356.docx]

Table S-1: Self-reported impacts on the caregiving environment

| Indicator | Name | Question |
| --- | --- | --- |
| $I_{1}$ | $H$ | Lack of information about how to provide care |
| $I_{2}$ | $I$ | Lack of or reduction of information about the person you care for |
| $I_{3}$ | $S$ | Loss or reduction of outside support |
| $I_{4}$ | $R$ | Loss or reduction of respite care such as day care facilities |
| $I_{5}$ | $V$ | Loss of visitation rights or access to a care facility |
| $I_{6}$ | $F$ | Not being able to provide face-to-face care if they live in their own home |
| $I_{7}$ | $W$ | Not being able to be with the person your care for during illness or death |
| $I_{8}$ | $G$ | Grieving alone or with limited support |
| $I_{9}$ | $E$ | Spending excessive time together |
| $I_{10}$ | $C$ | Being in a confined space together |
| $I_{11}$ | $P$ | Needing to wear protective clothing |

Notes: (1) This table shows the eleven environmental changes recorded in the CLIC Caregiver survey.

(2) Each of the binary indicators $I_{1},\cdots,I_{11}$ denotes the presence or absence of the given change.
