# Supplementary material for: The emotional cost of containment: a cross-sectional analysis of treatment effects among informal carers in South Asia during the COVID-19 pandemic
Source: Glob Health Action. 2025 Jun 3;18(1):2504227. doi: 10.1080/16549716.2025.2504227 (PMC12135087; doi:10.1080/16549716.2025.2504227)
Supplement: Table S4_Sample_statistics_CLEAN.docx [file ZGHA_A_2504227_SM8355.docx]

Table S-4: Sample statistics

| Panel A: Continuous Variables | |  | | |  | | | | |
| --- | --- | --- | --- | --- | --- | --- | --- | --- | --- |
| Variable | Sample Avg. (S.D.) (1) | Change in Burden Freq. Avg. (S.D.)  (2) | | | Pandemic-era Burden Freq. Avg. (S.D.)  (3) | | | | |
|  |  | Less Freq. | Same | More Freq. | Never | Rarely | Sometimes | Often | Always |
| **Change Dimensions** |  |  |  |  |  |  |  |  |  |
| $Trapped$ | 0.171 (0.287) | 0.067 (0.165) | 0.124 (0.250) | 0.253 (0.329) | 0.000 (0.000) | 0.000 (0.000) | 0.270 (0.291) | 0.554 (0.300) | 0.641 (0.341) |
| $NoInfo$ | 0.069 (0.216) | 0.039 (0.167) | 0.042 (0.176) | 0.109 (0.259) | 0.000 (0.000) | 0.000 (0.000) | 0.083 (0.217) | 0.262 (0.386) | 0.438 (0.403) |
| $NoConnect$ | 0.111 (0.223) | 0.096 (0.212) | 0.061 (0.151) | 0.172 (0.275) | 0.000 (0.000) | 0.000 (0.000) | 0.166 (0.250) | 0.341 (0.215) | 0.563 (0.338) |
| $NoVisit$ | 0.128 (0.334) | 0.154 (0.364) | 0.094 (0.292) | 0.160 (0.367) | 0.000 (0.000) | 0.000 (0.000) | 0.160 (0.367) | 0.452 (0.504) | 0.813 (0.403) |
| $PClothing$ | 0.156 (0.364) | 0.096 (0.298) | 0.094 (0.292) | 0.245 (0.431) | 0.000 (0.000) | 0.000 (0.000) | 0.196 (0.398) | 0.548 (0.504) | 1.000 (0.000) |
| **Control Variables** |  |  |  |  |  |  |  |  |  |
| *Age* | 33.49 (12.81) | 30.83 (11.94) | 33.19 (13.46) | 34.57 (12.21) | 28.75 (11.07) | 40.78 (13.54) | 33.24 (12.13) | 40.76 (11.75) | 37.13 (14.00) |
| *Number of Children* | 0.877 (1.214) | 0.789 (1.304) | 0.963 (1.342) | 0.803 (1.018) | 0.494 (0.895) | 1.631 (1.341) | 0.841 (1.122) | 1.333 (1.476) | 1.000 (1.317) |
| Panel B: Categorical Variables | |  |  |  |  |  |  |  |  |
| Variable | Sample $N$ (%)  (1) | Change in Burden Freq. $N$ (%)  (2) | | | Pandemic-era Burden Freq. $N$ (%).  (3) | | | | |
|  |  | Less Freq. | Same | More Freq. | Never | Rarely | Sometimes | Often | Always |
| **Care Partner Outcomes** |  |  |  |  |  |  |  |  |  |
| Pandemic burden |  |  |  |  |  |  |  |  |  |
| *Never* | 168 (37.0%) | 11 (2.4%) | 121 (26.7%) | 36 (7.9%) | 168 (37.0%) |  |  |  |  |
| *Rarely* | 65 (14.0%) | 18 (4.0%) | 32 (7.0%) | 15 (3.3%) |  | 65 (14.0%) |  |  |  |
| *Sometimes* | 163 (36.0%) | 20 (4.4%) | 54 (11.9%) | 89 (19.6%) |  |  | 163 (36.0%) |  |  |
| *Often* | 42 (9.0%) | 3 (0.6%) | 5 (1.1%) | 34 (7.6%) |  |  |  | 42 (9.0%) |  |
| *Always* | 16 (4.0%) | 0 (0.0%) | 2 (0.4%) | 14 (3.1%) |  |  |  |  | 16 (4.0%) |
| Change in Burden |  |  |  |  |  |  |  |  |  |
| *Less than Usual* | 52 (11.5%) | 52 (11.5%) |  |  | 25 (5.5%) | 8 (1.8%) | 16 (3.5%) | 1 (0.2%) | 2 (0.4%) |
| *Same as Usual* | 214 (47.1%) |  | 214 (47.1%) |  | 98 (21.6%) | 41 (9.0%) | 57 (12.6%) | 14 (3.1%) | 4 (0.9%) |
| *More than Usual* | 188 (41.4%) |  |  | 188 (41.4%) | 45 (9.9%) | 16 (3.5%) | 90 (19.8%) | 27 (6.0%) | 10 (2.2%) |
| **Primary stressors** |  |  |  |  |  |  |  |  |  |
| Recipient Condition |  |  |  |  |  |  |  |  |  |
| *Physical Condition* | 144 (31.7%) | 21 (4.6%) | 70 (15.4%) | 53 (11.7%) | 54 (11.9%) | 25 (5.5%) | 46 (10.1%) | 14 (3.1%) | 5 (1.1%) |
| *Dementia / Alzheimer’s* | 95 (20.9%) | 8 (1.7%) | 40 (8.8%) | 47 (10.3%) | 22 (4.8%) | 20 (4.4%) | 41 (0.9%) | 10 (2.2%) | 2 (0.4%) |
| *Other Condition* | 280 (61.7%) | 36 (7.9%) | 132 (29.1%) | 112 (24.7%) | 108 (23.8%) | 34 (7.5%) | 95 (21.0%) | 30 (6.6%) | 13 (2.9%) |
| Recipient Age |  |  |  |  |  |  |  |  |  |
| *Adult* | 374 (82.4%) | 35 (7.7%) | 176 (38.8%) | 163 (35.9%) | 148 (32.6%) | 49 (10.8%) | 130 (28.7%) | 37 (8.1%) | 10 (2.2%) |
| *Child (<18)* | 58 (12.8%) | 11 (2.4%) | 28 (6.2%) | 19 (4.2%) | 16 (3.5%) | 10 (2.2%) | 25 (5.5%) | 3 (0.7%) | 4 (0.9%) |
| *Adult and Child* | 22 (4.8%) | 6 (1.3%) | 10 (2.2%) | 6 (1.3%) | 4 (0.9%) | 6 (1.3%) | 8 (1.8%) | 2 (0.4%) | 2 (0.4%) |
| Recipient Relationship |  |  |  |  |  |  |  |  |  |
| *Spouse* | 28 (6.2%) | 2 (0.4%) | 16 (3.5%) | 10 (2.2%) | 5 (1.1%) | 12 (2.6%) | 8 (1.8%) | 3 (0.7%) | 0 (0.0%) |
| *Family* | 264 (58.1%) | 31 (6.8%) | 128 (28.2%) | 105 (23.1%) | 107 (23.6%) | 38 (8.4%) | 95 (20.9%) | 19 (4.2%) | 5 (1.1%) |
| *Relative* | 48 (10.6%) | 6 (1.3%) | 18 (4.0%) | 24 (5.3%) | 19 (4.2%) | 4 (0.9%) | 19 (4.2%) | 4 (0.9%) | 2 (0.4%) |
| *Non-relative* | 114 (25.1%) | 13 (2.9%) | 52 (11.5%) | 49 (10.8%) | 37 (8.1%) | 11 (2.4%) | 41 (9.0%) | 16 (3.5%) | 9 (2.0%) |
| Caregiving Venue |  |  |  |  |  |  |  |  |  |
| *Caregiver’s Home* | 240 (52.9%) | 26 (5.7%) | 115 (25.3%) | 99 (21.9%) | 98 (21.6%) | 43 (9.5%) | 80 (17.6%) | 17 (3.8%) | 2 (0.4%) |
| *Care Recipient’s Home* | 78 (17.2%) | 12 (2.6%) | 35 (7.7%) | 31 (6.8%) | 25 (5.5%) | 6 (1.3%) | 36 (7.9%) | 7 (1.5%) | 4 (0.9%) |
| *Care facility* | 51 (11.2%) | 5 (1.1%) | 23 (5.1%) | 23 (5.1%) | 12 (2.6%) | 13 (2.9%) | 20 (4.4%) | 2 (0.4%) | 4 (0.9%) |
| *Other* | 85 (18.7%) | 9 (2.0%) | 41 (9.0%) | 35 (7.7%) | 33 (7.3%) | 3 (0.7%) | 27 (6.0%) | 16 (3.5%) | 6 (1.3%) |
| **Secondary role strains/concordances** | |  |  |  |  |  |  |  |  |
| Financial Status |  |  |  |  |  |  |  |  |  |
| *Poor* | 64 (14.1%) | 13 (2.9%) | 21 (4.6%) | 30 (6.6%) | 23 (5.1%) | 6 (1.3%) | 30 (6.6%) | 5 (1.1%) | 0 (0.0%) |
| *Fairly Good* | 276 (60.8%) | 28 (6.2%) | 129 (28.4%) | 119 (26.2%) | 101 (22.2%) | 41 (9.0%) | 97 (21.4%) | 28 (6.2%) | 9 (2.0%) |
| *Very Good* | 114 (25.1%) | 11 (2.4%) | 64 (14.1%) | 39 (8.6%) | 44 (9.7%) | 18 (4.0%) | 36 (7.9%) | 9 (2.0%) | 7 (1.5%) |
| Physical Health |  |  |  |  |  |  |  |  |  |
| *Poor* | 9 (2.0%) | 1 (0.2%) | 3 (0.7%) | 5 (1.1%) | 2 (0.4%) | 0 (0.0%) | 5 (1.1%) | 2 (0.4%) | 0 (0.0%) |
| *Fair* | 53 (11.7%) | 5 (1.1%) | 18 (4.0%) | 30 (6.6%) | 19 (4.2%) | 4 (0.9%) | 27 (6.0%) | 2 (0.4%) | 1 (0.2%) |
| *Good* | 179 (39.4%) | 19 (4.2%) | 81 (17.8%) | 79 (17.4%) | 60 (13.2%) | 27 (6.0%) | 70 (15.4%) | 17 (3.8%) | 5 (1.1%) |
| *Very Good* | 158 (34.8%) | 22 (4.8%) | 79 (17.4%) | 57 (12.6%) | 63 (13.9%) | 23 (5.1%) | 45 (9.9%) | 19 (4.2%) | 8 (1.8%) |
| *Excellent* | 55 (12.1%) | 5 (1.1%) | 33 (7.3%) | 17 (3.7%) | 24 (5.3%) | 11 (2.4%) | 16 (3.5%) | 2 (0.4%) | 2 (0.4%) |
| Mental Health |  |  |  |  |  |  |  |  |  |
| *Poor* | 15 (3.3%) | 1 (0.2%) | 4 (0.9%) | 10 (2.2%) | 5 (1.1%) | 0 (0.0%) | 8 (1.8%) | 2 (0.4%) | 0 (0.0%) |
| *Fair* | 59 (13.0%) | 10 (2.2%) | 21 (4.6%) | 28 (6.2%) | 20 (4.4%) | 7 (1.5%) | 22 (4.8%) | 8 (1.8%) | 2 (0.4%) |
| *Good* | 173 (38.1%) | 14 (3.1%) | 83 (18.3%) | 76 (16.8%) | 65 (14.3%) | 22 (4.8%) | 65 (14.3%) | 16 (3.5%) | 5 (1.1%) |
| *Very Good* | 129 (28.4%) | 15 (3.3%) | 66 (14.5%) | 48 (10.6%) | 49 (10.8%) | 17 (3.8%) | 44 (9.7%) | 13 (2.9%) | 6 (1.3%) |
| *Excellent* | 78 (17.2%) | 12 (2.6%) | 40 (8.8%) | 26 (5.7%) | 29 (6.4%) | 19 (4.2%) | 24 (5.3%) | 3 (0.7%) | 3 (0.7%) |
| Employment |  |  |  |  |  |  |  |  |  |
| *Student/Unemployed* | 181 (40.0%) | 24 (5.3%) | 93 (20.5%) | 64 (14.1%) | 84 (18.6%) | 19 (4.2%) | 64 (14.1%) | 10 (2.2%) | 4 (0.9%) |
| *Employed* | 220 (48.0%) | 19 (4.2%) | 94 (20.7%) | 107 (23.6%) | 70 (15.4%) | 37 (8.1%) | 79 (17.4%) | 22 (4.8%) | 12 (2.6%) |
| *Homemaker* | 27 (6.0%) | 5 (1.1%) | 15 (3.3%) | 7 (1.5%) | 8 (1.8%) | 5 (1.1%) | 10 (2.2%) | 4 (0.9%) | 0 (0.0%) |
| *Other* | 26 (6.0%) | 4 (0.9%) | 12 (2.6%) | 10 (2.2%) | 6 (1.3%) | 4 (0.9%) | 10 (2.2%) | 6 (1.3%) | 0 (0.0%) |
| Education |  |  |  |  |  |  |  |  |  |
| *Primary* | 41 (9.0%) | 4 (0.9%) | 29 (6.4%) | 8 (1.8%) | 11 (2.4%) | 8 (1.8%) | 13 (2.9%) | 8 (1.8%) | 1 (0.2%) |
| *Secondary* | 69 (15.2%) | 10 (2.2%) | 41 (9.0%) | 18 (4.0%) | 38 (8.4%) | 4 (0.9%) | 24 (5.3%) | 3 (0.6%) | 0 (0.0%) |
| *Post-Secondary* | 344 (75.8%) | 38 (8.4%) | 144 (31.7%) | 162 (35.6%) | 119 (26.2%) | 53 (11.7%) | 126 (27.7%) | 31 (6.8%) | 15 (3.3%) |
| **Background/context Variables** | |  |  |  |  |  |  |  |  |
| Gender |  |  |  |  |  |  |  |  |  |
| *Female* | 273 (60.1%) | 31 (6.8%) | 126 (27.7%) | 116 (25.6%) | 107 (23.6%) | 35 (7.7%) | 100 (22.0%) | 24 (5.3%) | 7 (1.5%) |
| *Male* | 181 (39.9%) | 21 (4.6%) | 88 (19.4%) | 72 (15.9%) | 61 (13.4%) | 30 (6.6%) | 63 (13.9%) | 18 (4.0%) | 9 (2.0%) |
| Marital Status |  |  |  |  |  |  |  |  |  |
| *Single/unmarried* | 210 (46.3%) | 30 (6.6%) | 102 (22.5%) | 78 (17.2%) | 119 (26.2%) | 0 (0.0%) | 71 (15.6%) | 12 (2.6%) | 8 (1.8%) |
| *Married* | 227 (50.0%) | 20 (4.4%) | 102 (22.5%) | 105 (23.1%) | 49 (10.8%) | 56 (12.3%) | 84 (18.5%) | 30 (6.6%) | 8 (1.8%) |
| *Other* | 17 (3.7%) | 2 (0.4%) | 10 (2.2%) | 5 (1.1%) | 0 (0.0%) | 9 (2.0%) | 8 (1.8%) | 0 (0.0%) | 0 (0.0%) |
| National Origin |  |  |  |  |  |  |  |  |  |
| *Bangladesh* | 123 (27.0%) | 7 (1.5%) | 61 (13.4%) | 55 (12.1%) | 43 (9.5%) | 1 (0.2%) | 49 (10.8%) | 25 (5.5%) | 5 (1.1%) |
| *India* | 116 (26.0%) | 8 (1.8%) | 46 (10.1%) | 62 (13.8%) | 33 (7.3%) | 29 (6.4%) | 40 (8.8%) | 8 (1.8%) | 6 (1.3%) |
| *Pakistan* | 215 (47.0%) | 37 (8.1%) | 107 (23.6%) | 71 (15.6%) | 92 (20.2%) | 35 (7.7%) | 74 (16.3%) | 9 (2.0%) | 5 (1.1%) |
| Residential setting |  |  |  |  |  |  |  |  |  |
| *City* | 278 (61.2%) | 35 (7.7%) | 128 (28.1%) | 115 (25.3%) | 110 (24.3%) | 36 (7.9%) | 105 (23.1%) | 17 (3.7%) | 10 (2.2%) |
| *Town* | 129 (28.4%) | 14 (3.1%) | 62 (13.8%) | 53 (11.7%) | 43 (9.5%) | 20 (4.4%) | 40 (8.8%) | 21 (4.6%) | 5 (1.1%) |
| *Village* | 47 (10.4%) | 3 (0.6%) | 24 (5.3%) | 20 (4.4%) | 15 (3.3%) | 9 (2.0%) | 18 (4.0%) | 4 (0.9%) | 1 (0.2%) |

Notes: (1) The sample describes of 454 care partners born and living in Bangladesh, India, and Pakistan.

(2) A care partner was a respondent who answered "Yes" to the question, "Do you provide care and support to a family member or friend with a long-term or life-limiting health problem or disability (including mental health).
