# Supplementary material for: The emotional cost of containment: a cross-sectional analysis of treatment effects among informal carers in South Asia during the COVID-19 pandemic
Source: Glob Health Action. 2025 Jun 3;18(1):2504227. doi: 10.1080/16549716.2025.2504227 (PMC12135087; doi:10.1080/16549716.2025.2504227)
Supplement: Table S5_Empirical_tests.docx [file ZGHA_A_2504227_SM8354.docx]

Table S-5: Four empirical tests

|  | | |
| --- | --- | --- |
| Pandemic burden frequency | | |
| Level | Effect | Sig. |
| Never | -0.598 (0.166) | ^**^ |
| Rarely | -0.019 (0.020) |  |
| Sometimes | 0.384 (0.112) | ^**^ |
| Frequently | 0.188 (0.061) | ^**^ |
| Always | 0.045 (0.025) |  |
| Never | -0.223 (0.469) |  |
| Rarely | -0.014 (0.031) |  |
| Sometimes | 0.179 (0.377) |  |
| Frequently | 0.042 (0.091) |  |
| Always | 0.015 (0.036) |  |
| Never | -0.368 (0.185) | ^*^ |
| Rarely | -0.011 (0.015) |  |
| Sometimes | 0.262 (0.133) | ^*^ |
| Frequently | 0.095 (0.051) |  |
| Always | 0.023 (0.017) |  |
| Never | -0.299 (0.102) | ^**^ |
| Rarely | -0.006 (0.011) |  |
| Sometimes | 0.220 (0.075) | ^**^ |
| Frequently | 0.078 (0.031) | ^*^ |
| Always | 0.007 (0.007) |  |
| Never | -0.362 (0.132) | ^**^ |
| Rarely | 0.000 (0.014) |  |
| Sometimes | 0.248 (0.095) | ^**^ |
| Frequently | 0.106 (0.040) | ^**^ |
| Always | 0.008 (0.008) |  |
|  | | |
| Pandemic burden frequency | | |
| Level | Effect | Sig. |
| Never | -0.391 (0.162) | ^*^ |
| Rarely | -0.070 (0.036) |  |
| Sometimes | 0.217 (0.099) | ^*^ |
| Frequently | 0.198 (0.088) | ^*^ |
| Always | 0.045 (0.031) |  |
| Never | 0.246 (0.571) |  |
| Rarely | 0.006 (0.018) |  |
| Sometimes | -0.185 (0.432) |  |
| Frequently | -0.037 (0.086) |  |
| Always | -0.030 (0.074) |  |
| Never | -0.436 (0.195) | ^*^ |
| Rarely | -0.001 (0.022) |  |
| Sometimes | 0.321 (0.145) | ^*^ |
| Frequently | 0.065 (0.038) |  |
| Always | 0.051 (0.035) |  |
| Never | -0.412 (0.129) | ^**^ |
| Rarely | -0.041 (0.027) |  |
| Sometimes | 0.381 (0.121) | ^**^ |
| Frequently | 0.072 (0.048) |  |
| Always | ----------------- |  |
| Never | -0.341 (0.155) | ^*^ |
| Rarely | -0.024 (0.080) |  |
| Sometimes | 0.221 (0.113) |  |
| Frequently | 0.145 (0.069) | ^*^ |
| Always | ---------------- |  |

| Panel A: Basic treatment effects (Test 1) | | | | |
| --- | --- | --- | --- | --- |
|  |  | Change in burden frequency | | |
| Treatment | Sample | Level | Effect | Sig. |
| $Trapped$ | 43 treated  233 controls | less often | -0.332 (0.120) | ^**^ |
|  |  | the same | -0.247 (0.087) | ^**^ |
|  |  | more often | 0.578 (0.185) | ^**^ |
| $NoInfo$ | 4 treated  77 controls | less often | -0.826 (0.323) | ^*^ |
|  |  | the same | -0.068 (0.223) |  |
|  |  | more often | 0.895 (0.305) | ^**^ |
| $NoConnect$ | 27 treated  233 controls | less often | -0.273 (0.139) | ^*^ |
|  |  | the same | -0.156 (0.084) |  |
|  |  | more often | 0.429 (0.210) | ^*^ |
| $NoVisit$ | 13 treated  233 controls | less often | -0.022 (0.074) |  |
|  |  | the same | -0.011 (0.038) |  |
|  |  | more often | 0.033 (0.111) |  |
| $PClothing$ | 8 treated  233 controls | less often | -0.097 (0.106) |  |
|  |  | the same | -0.052 (0.056) |  |
|  |  | more often | 0.148 (0.160) |  |
| Panel B: Propensity Score Matching (Test 2) | | | | |
|  |  | Change in burden frequency | | |
| Treatment | Sample | Level | Effect | Sig. |
| $Trapped$ | 43 treated  118 controls | less often | -0.247 (0.115) | ^*^ |
|  |  | the same | -0.218 (0.094) | ^*^ |
|  |  | more often | 0.464 (0.193) | ^*^ |
| $NoInfo$ | 4 treated  42 controls | less often | -0.401 (0.250) |  |
|  |  | the same | -0.012 (0.178) |  |
|  |  | more often | 0.413 (0.257) |  |
| $NoConnect$ | 27 treated  108 controls | less often | -0.340 (0.175) |  |
|  |  | the same | -0.099 (0.074) |  |
|  |  | more often | 0.439 (0.206) | ^*^ |
| $NoVisit$ | 13 treated  54 controls | less often | -0.053 (0.084) |  |
|  |  | the same | -0.018 (0.031) |  |
|  |  | more often | 0.071 (0.111) |  |
| $PClothing$ | 8 treated  25 controls | less often | -0.119 (0.110) |  |
|  |  | the same | -0.070 (0.078) |  |
|  |  | more often | 0.190 (0.169) |  |

Table S-5, continued

|  | | | | |
| --- | --- | --- | --- | --- |
| Pandemic burden frequency | | | | |
| Level | | Effect | | Sig. |
| Never | | -0.365 (0.145) | | ^*^ |
| Rarely | | -0.076 (0.042) | |  |
| Sometimes | | 0.158 (0.073) | | ^*^ |
| Frequently | | 0.206 (0.095) | | ^*^ |
| Always | | 0.076 (0.056) | |  |
| Never | | 0.427 (0.459) | |  |
| Rarely | | 0.124 (0.184) | |  |
| Sometimes | | -0.395 (0.467) | |  |
| Frequently | | -0.034 (0.076) | |  |
| Always | | -0.122 (0.164) | |  |
| Never | | -0.259 (0.125) | | ^*^ |
| Rarely | | -0.030 (0.021) | |  |
| Sometimes | | 0.167 (0.087) | |  |
| Frequently | | 0.053 (0.034) | |  |
| Always | | 0.069 (0.037) | |  |
| Never | | -0.410 (0.083) | | ^**^ |
| Rarely | | -0.209 (0.142) | |  |
| Sometimes | | 0.550 (0.177) | | ^**^ |
| Frequently | | 0.069 (0.053) | |  |
| Always | | ----------------- | |  |
| Never | | -0.047 (0.030) | |  |
| Rarely | | -0.024 (0.015) | |  |
| Sometimes | | -0.012 (0.011) | |  |
| Frequently | | 0.063 (0.040) | |  |
| Always | | 0.021 (0.012) | |  |
|  | | | | |
| Pandemic burden frequency | | | | |
| Averages | | | $t$ | Sig. |
| Base | Treated | |  |  |
| 2.367 | 2.721 | | 1.862 | ^*^ |
| 2.033 | 2.250 | | 0.364 |  |
| 1.911 | 2.556 | | 2.767 | ^**^ |
| 2.015 | 2.846 | | 3.690 | ^**^ |
| 2.775 | 2.625 | | -0.728 |  |

| Panel C: Propensity Score Matching and Entropy Balancing (Test 3) | | | | | | | |
| --- | --- | --- | --- | --- | --- | --- | --- |
|  |  | Change in burden frequency | | | | | |
| Treatment | Sample | Level | | Effect | | | Sig. |
| $Trapped$ | 43 treated  118 controls | less often | | -0.173 (0.072) | | | ^*^ |
|  |  | the same | | -0.298 (0.098) | | | ^**^ |
|  |  | more often | | 0.471 (0.160) | | | ^**^ |
| $NoInfo$ | 4 treated  42 controls | less often | | -0.241 (0.134) | | |  |
|  |  | the same | | -0.074 (0.163) | | |  |
|  |  | more often | | 0.315 (0.220) | | |  |
| $NoConnect$ | 27 treated  108 controls | less often | | -0.303 (0.130) | | | ^*^ |
|  |  | the same | | -0.131 (0.091) | | |  |
|  |  | more often | | 0.435 (0.186) | | | ^*^ |
| $NoVisit$ | 13 treated  54 controls | less often | | -0.088 (0.051) | | |  |
|  |  | the same | | -0.030 (0.036) | | |  |
|  |  | more often | | 0.118 (0.062) | | |  |
| $PClothing$ | 8 treated  25 controls | less often | | -0.062 (0.036) | | |  |
|  |  | the same | | -0.041 (0.022) | | |  |
|  |  | more often | | 0.102 (0.056) | | |  |
| Panel D: Paired $t$-tests (Test 4) | | | | | | | |
|  |  |  | Change in burden frequency | | | | |
| Treatment | Sample | *df* | Averages | | | $t$ | Sig. |
|  |  |  | Base | | Treated |  |  |
| $Trapped$ | 43 treated  118 controls | 42 | 2.242 | | 2.535 | 3.087 | ^**^ |
| $NoInfo$ | 4 treated  42 controls | 3 | 1.983 | | 2.250 | 1.089 |  |
| $NoConnect$ | 27 treated  108 controls | 26 | 2.044 | | 2.333 | 2.055 | ^*^ |
| $NoVisit$ | 13 treated  54 controls | 23 | 2.046 | | 2.154 | 0.514 |  |
| $PClothing$ | 8 treated  25 controls | 7 | 2.500 | | 2.625 | 0.459 |  |

Notes: (1) The table presents the effects of the treatments on change in burden frequency (left column) and pandemic burden frequency (right column).

(2) Each treatment effect is based on a comparison of treated observations and control observations. The treated observations were those for which the specified treatment was positive, but all other treatments were zero. The control observations were those for which all treatments were zero. The basic treatment effects employed the whole control sample, consisting of 233 observations. The enhanced treatment effects and paired $t$-tests used the propensity score matched observations, a subset of the control sample.

(3) Propensity score matching was carried out with the Mahalanobis distance measure. For each treated observation, five matching controls were identified using nearest neighbor matching. For further details, consult the Supplementary appendix.

(4) The $t$-test results were obtained from the same subsamples employed in propensity-matched treatment effects analysis. For each factor, burden change (burden level) among treated carers was hypothesized to exceed the baseline level (the averaged impact among matching observations). The outputs were obtained through one-tailed paired $t$-tests of these conjectures.

(5) The second treatment, $NoInfo$, was a special case. Because of data limitations, there were only four treated observations, all from Pakistan. Therefore, in the basic treatment effects analysis, instead of the whole control sample, the $77$ control observations from Pakistan were used. Furthermore, in the enhanced treatment effects analysis, instead of five elements, ten matching elements (all from the Pakistani control sample) were used to enlarge the sample size.

(6) In Panels B and C, the *Always* effects of the $NoVisit$ treatment are missing because the matched sample included no observations at this level. Likewise, in Panel B, the *Always* effect of the $PClothing$ treatment is missing for the same reason.

(7) For each treatment effect ($t$ value), one star denotes a $p$-value between 0.01 and 0.05; two stars, a $p$-value less than 0.01.
