# Supplementary material for: The emotional cost of containment: a cross-sectional analysis of treatment effects among informal carers in South Asia during the COVID-19 pandemic
Source: Glob Health Action. 2025 Jun 3;18(1):2504227. doi: 10.1080/16549716.2025.2504227 (PMC12135087; doi:10.1080/16549716.2025.2504227)
Supplement: Figure S1_Smoothed Treatment 2 Effect Curves.docx [file ZGHA_A_2504227_SM8353.docx]

| 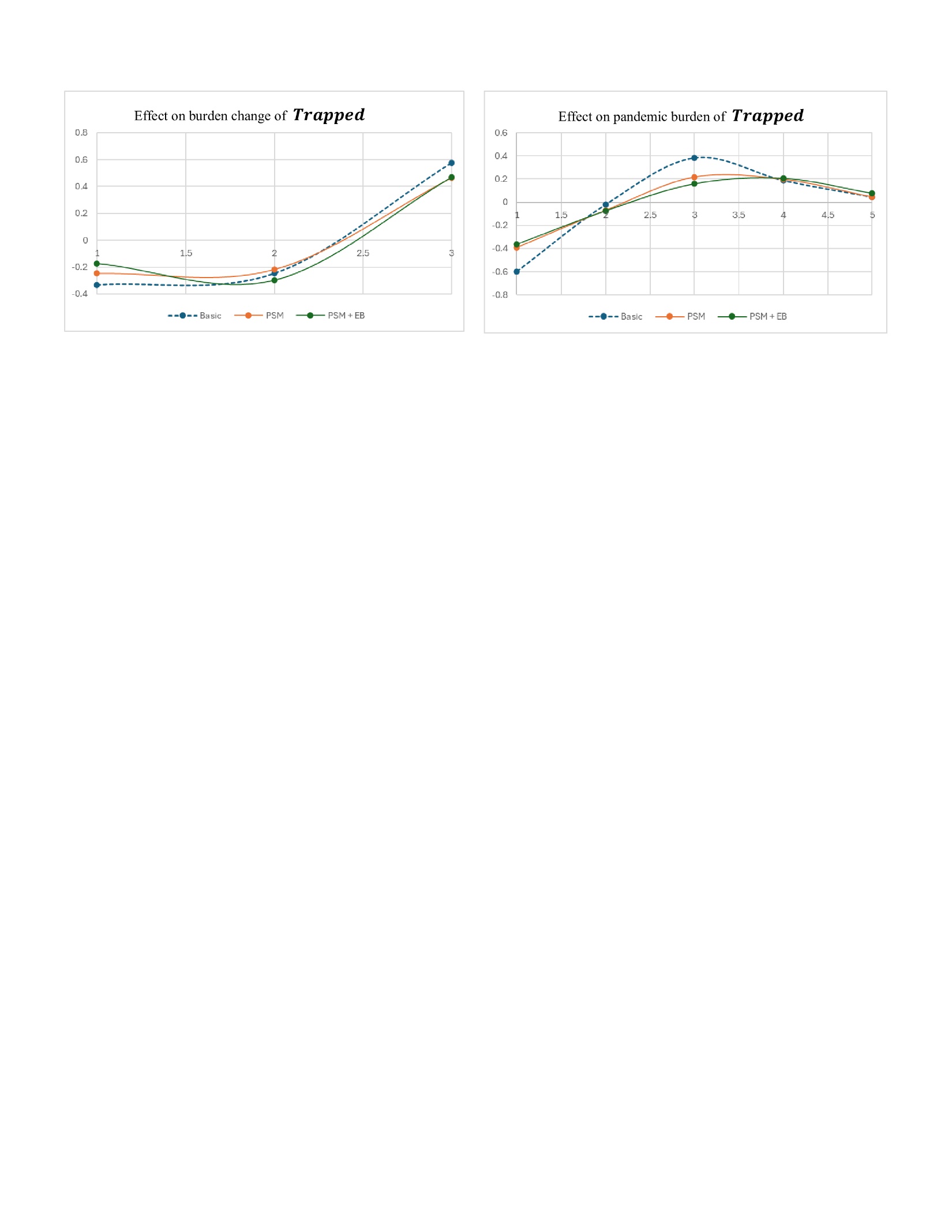 |
| --- |
| 1. Treatment effects of ***Trapped*** on burden change and pandemic burden |
| 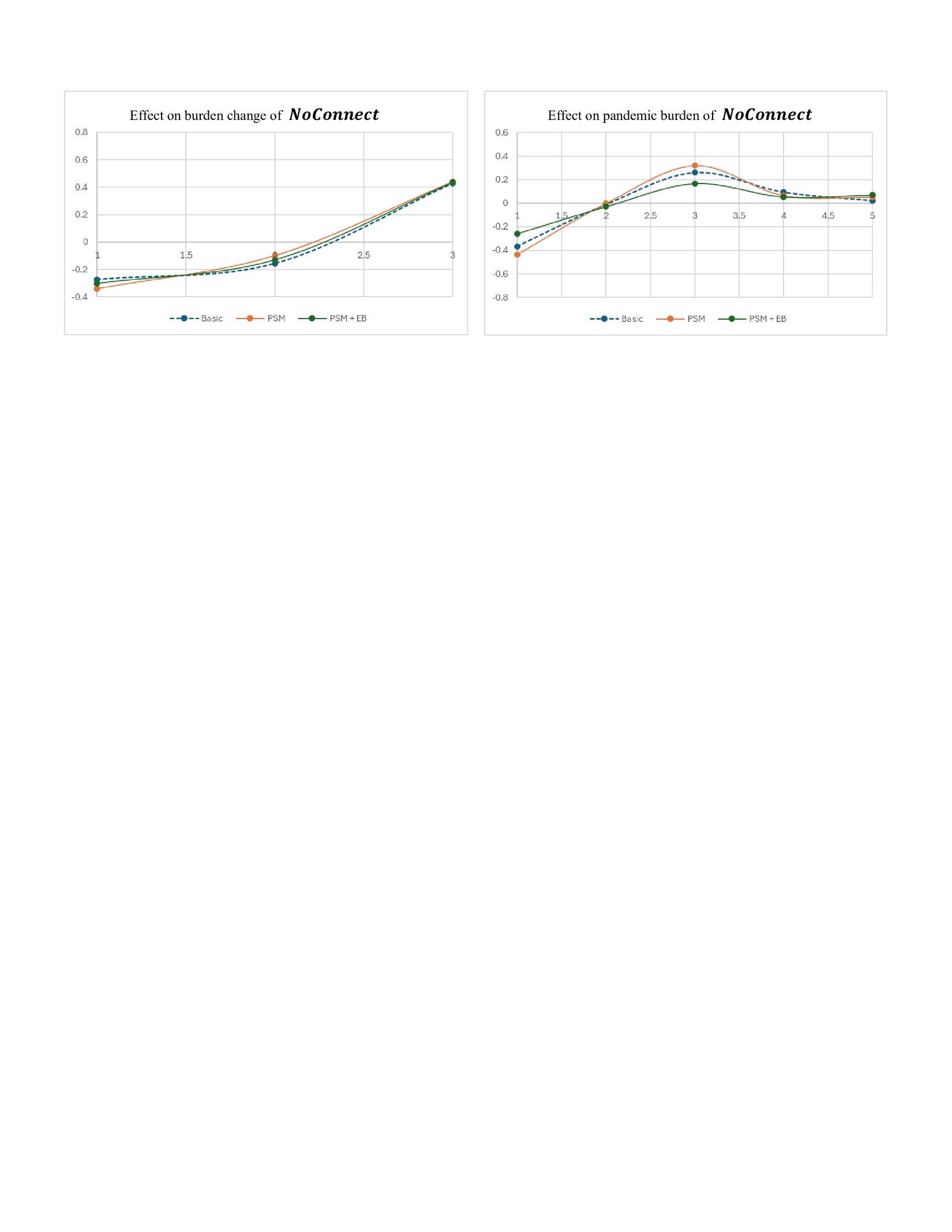 |
| 1. Treatment effects of ***NoConnect*** on burden change and pandemic burden |
| 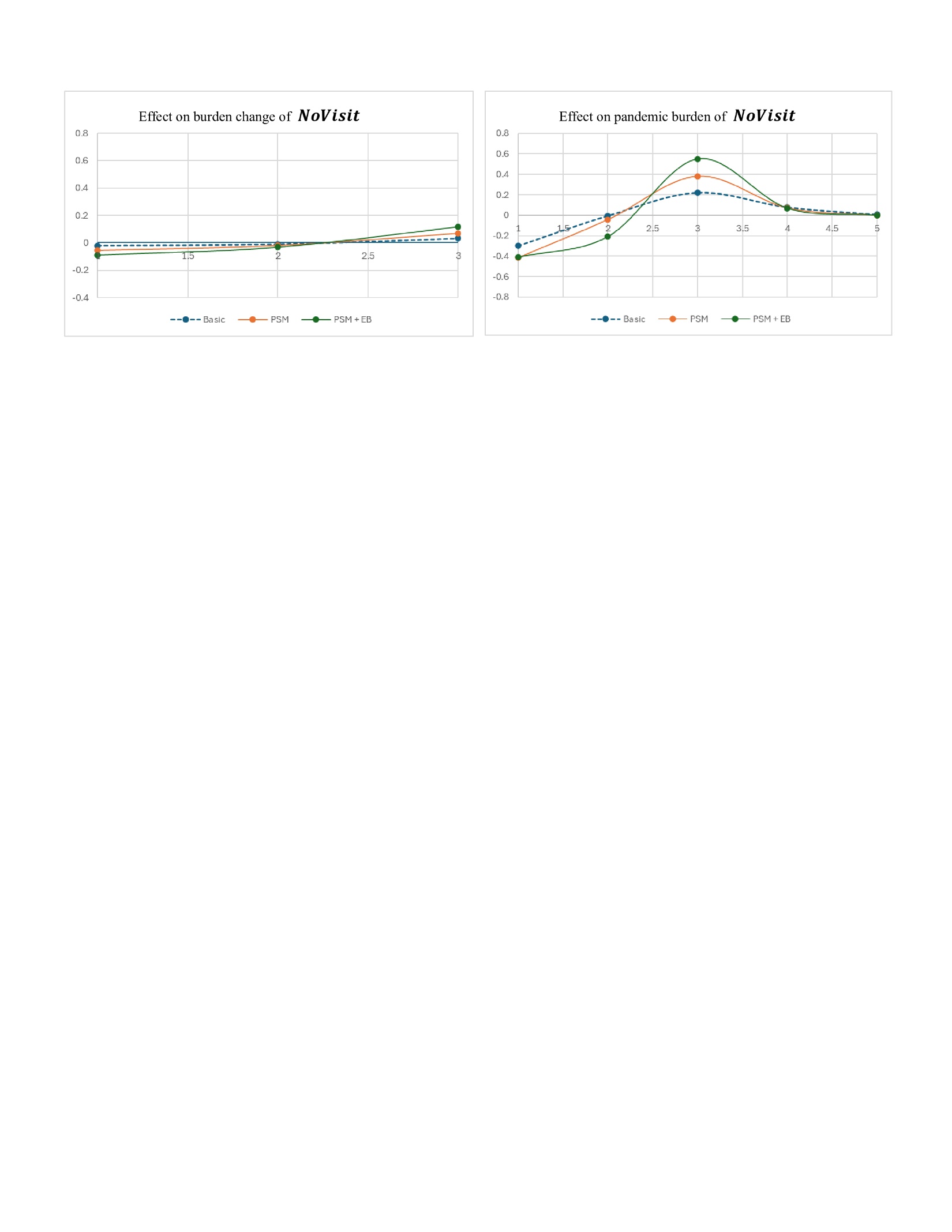 |
| 1. Treatment effect of ***NoVisit*** on burden change and pandemic burden |

Figure S-1: Smoothed treatment effect curves

Note: In each diagram, the three sets of treatment effects (basic, propensity score matched, and propensity score matched with entropy balancing) are plotted. Smoothed treatment effect lines are used to connect the plotted values.
